# Assessment of Adherence to Reporting Guidelines by Commonly Used Clinical Prediction Models From a Single Vendor: A Systematic Review

**DOI:** 10.1001/jamanetworkopen.2022.27779

**Published:** 2022-08-19

**Authors:** Jonathan H. Lu, Alison Callahan, Birju S. Patel, Keith E. Morse, Dev Dash, Michael A. Pfeffer, Nigam H. Shah

**Affiliations:** 1 Center for Biomedical Informatics Research, Stanford University School of Medicine, Stanford, California; 2 Department of Pediatrics, Stanford University School of Medicine, Stanford, California; 3 Department of Clinical Informatics, Lucile Packard Children’s Hospital, Palo Alto, California; 4 Technology and Digital Solutions, Stanford Medicine, Stanford, California; 5 Clinical Excellence Research Center, Stanford Medicine, Stanford, California

## Abstract

**Importance:**

Various model reporting guidelines have been proposed to ensure clinical prediction models are reliable and fair. However, no consensus exists about which model details are essential to report, and commonalities and differences among reporting guidelines have not been characterized. Furthermore, how well documentation of deployed models adheres to these guidelines has not been studied.

**Objectives:**

To assess information requested by model reporting guidelines and whether the documentation for commonly used machine learning models developed by a single vendor provides the information requested.

**Evidence Review:**

MEDLINE was queried using *machine learning model card* and *reporting machine learning* from November 4 to December 6, 2020. References were reviewed to find additional publications, and publications without specific reporting recommendations were excluded. Similar elements requested for reporting were merged into representative items. Four independent reviewers and 1 adjudicator assessed how often documentation for the most commonly used models developed by a single vendor reported the items.

**Findings:**

From 15 model reporting guidelines, 220 unique items were identified that represented the collective reporting requirements. Although 12 items were commonly requested (requested by 10 or more guidelines), 77 items were requested by just 1 guideline. Documentation for 12 commonly used models from a single vendor reported a median of 39% (IQR, 37%-43%; range, 31%-47%) of items from the collective reporting requirements. Many of the commonly requested items had 100% reporting rates, including items concerning outcome definition, area under the receiver operating characteristics curve, internal validation, and intended clinical use. Several items reported half the time or less related to reliability, such as external validation, uncertainty measures, and strategy for handling missing data. Other frequently unreported items related to fairness (summary statistics and subgroup analyses, including for race and ethnicity or sex).

**Conclusions and Relevance:**

These findings suggest that consistent reporting recommendations for clinical predictive models are needed for model developers to share necessary information for model deployment. The many published guidelines would, collectively, require reporting more than 200 items. Model documentation from 1 vendor reported the most commonly requested items from model reporting guidelines. However, areas for improvement were identified in reporting items related to model reliability and fairness. This analysis led to feedback to the vendor, which motivated updates to the documentation for future users.

## Key Points

**Question:**

What items are collectively requested by model reporting guidelines and does documentation for deployed clinical predictive models report this information?

**Findings:**

This systematic review combined 15 model reporting guidelines and identified 220 distinct requested items. A review of the documentation of 12 deployed models from a single vendor found that the median item completion rate was 39%, and although commonly requested items were highly reported, at least half of the documentation could have provided more information on reliability (including external validation) and fairness.

**Meaning:**

These findings suggest that items collectively requested by guidelines represent a substantial reporting burden, and additions regarding reliability and fairness may improve their documentation.

## Teaser

This systematic review summarizes clinical predictive model reporting guidelines, characterizes how often items are requested across guidelines, and assesses whether the documentation for commonly deployed models provides the information requested by model reporting guidelines.

## Introduction

Despite good predictive performance in metrics such as the area under the receiver operating characteristic (AUROC) curve, the use of machine learning models trained on electronic health record data^1^ to guide care has not often been demonstrated to translate into measurable clinical gains in the form of better medical care, lower cost, or more equitable outcomes,^2,3,4^ leading to a gap that has been referred to as an “artificial intelligence (AI) chasm.”^5^ Some potential reasons for this chasm are that current models are not useful,^4,6,7^ reliable,^8,9^ or fair.^10,11,12,13,14,15,16,17,18^ Nevertheless, predictive models have frequently been deployed in health care settings without transparency or independent validation,^19,20^ and their subsequent failures have occasionally been met with public outcry.^2,21,22,23^

Adhering to model reporting guidelines is one way to improve the reliability,^24,25,26,27,28^ fairness,^29,30^ and usefulness^25,31,32,33,34^ of clinical predictive models. Reporting guidelines have long been used to assess the strength of clinical trial,^35,36^ observational,^37^ and diagnostic^38^ studies. Guidelines about reporting the performance of predictive models are receiving increasing attention, including from the National Institutes of Health,^39^ and several more guidelines are in development.^40,41,42^

However, limited information is available about the overlapping coverage of these varying guidelines, making it difficult for participants in the community to understand what common set of items should be expected, let alone which items can be reported in practice. As a result, important information is often missing from documentation. For example, a review that examined 164 models described in the scientific literature^43^ found low reporting rates of demographic variables such as race (36%) and socioeconomic status (8%) as well as low external validation rates (12%). A critical review of published models for diagnosis and prognosis of COVID-19^44^ found that most models were at high risk of bias due to poor reporting.

The goal of this systematic review was to summarize clinical predictive model reporting guidelines and characterize how often items are requested across guidelines. In addition, we assessed whether the documentation for commonly deployed models provided the information requested by model reporting guidelines. Compared with previous work,^43,44^ we focused on user-facing product documentation accompanying models, which allowed us to analyze models that have been deployed in practice and are not limited to those described in peer-reviewed publications. Furthermore, we comprehensively measured the reporting rates of every requested item covered in all the guidelines.

## Methods

Our analysis consisted of 2 phases. We first compiled model reporting guidelines and summarized them to identify the unique reporting items they request and analyzed the items that are the most and least requested across all guidelines. A team of 4 reviewers (J.H.L., A.C., B.S.P., and K.E.M.) and 1 adjudicator (D.D.) then assessed a sample of model documentation to identify the items they report as well as any gaps in reporting. We describe each of these phases in detail and provide additional information in the eMethods in the Supplement. Through the review process we addressed the items from the Preferred Reporting Items for Systematic Reviews and Meta-analyses (PRISMA) reporting guideline that were applicable to this study.

### Summarizing Model Reporting Guidelines

We searched MEDLINE via PubMed using queries for *machine learning model card* and *reporting machine learning* from November 4 to December 6, 2020. We reviewed citations to find additional publications. Finally, we excluded publications that did not give specific model reporting recommendations.

We then gathered the set of reportable elements in these reporting guidelines and merged similar elements into distinct, representative items to eliminate duplication. For example, “report the intended user of the model”^31^ and “describe external validation strategy”^24^ are unique items. First, we identified an initial set of elements by reviewing each reporting guideline, including the explanation and elaboration documents and AI extensions to verify that every guideline’s elements were captured. Second, we reviewed each element and, using expert judgment, merged those that requested the same information into the same item. We recorded each study’s phrases describing the elements to enable a full traceback of which elements were merged into each item. Last, we created a 1-line summary of each item to share for reviewers to reference (eAppendix in the Supplement).

### Assessing Item Reporting in Existing Model Documentation

To assess the use of this collective set of reportable items in user-facing documentation, we obtained a convenience sample of model documentation in March 2021. We reviewed the user-facing documentation (analogous to a drug package insert) provided by 1 vendor (Epic Systems Corporation), which they term *cognitive computing model briefs* (hereafter referred to as model briefs) (eTable 1 in the Supplement). Each model brief has a community adoption score that represents the proportion of organizations that have used a specific model of organizations using any model and takes values from a scale ranging from 1 to 3. We chose all models that had a community adoption score of 2 or 3 in March 2021. The model briefs with community adoption score of 3 of 3 were the Deterioration Index,^45^ Early Detection of Sepsis,^46^ Risk of Unplanned Readmission (Version 2),^47^ Risk of Patient No-Show (Version 2),^48^ Pediatric Hospital Admissions and ED Visits,^49^ and Risk of Hospital Admission or ED Visit (Version 2)^50^ models. The model briefs with community adoption of 2 of 3 were for Inpatient Risk of Falls,^51^ Projected Block Utilization,^52^ Remaining Length of Stay,^53^ Hospital Admissions for Heart Failure,^54^ Hospital Admissions and ED Visits for Asthma,^55^ and Hypertension.^56^ Note that model briefs are periodically updated by the vendor, and we assessed the most recent version available at the time of our study.

The 4 reviewers read each of the 12 model briefs and independently assessed whether they reported information specified in the items as summarized in the eAppendix in the Supplement (process described in the eMethods in the Supplement). Specifically, for each item, each reviewer first determined whether the item was applicable to the model, and if it was determined to be applicable, whether that item was reported or not reported. For example, an item such as “a link to the clinical trial registration” was determined to be not applicable to models where documentation does not intend to describe a clinical trial. The reviewers’ specific assessments are all available (eAppendix in the Supplement). The reviewer then decided whether the model brief reported the information requested in the item, recording the relevant part of the model brief supporting their decision. Reviewers were informatics experts (J.H.L. and A.C.) and clinicians (B.J.P. and K.E.M.) who had expertise in deployment of machine learning at our academic medical center.

The adjudicator (D.D.) then reviewed the items for which there was disagreement among reviewers to make a final determination. The adjudicator was constrained to choose only from the options already selected by the reviewers. The adjudicator was also a clinician with similar expertise in deployment of machine learning models. Detailed terminology and summary statistics calculation are provided in the eMethods in the Supplement.

## Results

### Items Requested by Model Reporting Guidelines

The literature search for model reporting guidelines resulted in a list of 27 publications,^25,29,30,31,32,33,34,38,41,57,58,59,60,61,62,63,64,65,66,67,68,69,70,71,72,73,74^ and a citation review yielded 3 additional publications.^26,27,28^ We excluded publications that did not provide specific model reporting recommendations, yielding 15 model reporting guidelines (Table 1).^24,25,26,27,28,29,30,31,32,33,34,35,57,58,59,74,75,76,77,78,79,80^

**Table 1.** Summary of 15 Model Reporting Guideline Papers^a^

Source	Abbreviation or short title	Title	Journal	Total No. of citations^b^	Items^c^
Schulz et al,^35^ 2010	CONSORT-AI	CONSORT 2010 statement: updated guidelines for reporting parallel group randomised trials	*International Journal of Surgery*	11 529	68
Moher et al,^78^ 2010		CONSORT 2010 Explanation and Elaboration: updated guidelines for reporting parallel group randomized trials	*Journal of Clinical Epidemiology*		
Liu et al,^32^ 2020		Reporting guidelines for clinical trial reports for interventions involving artificial intelligence: the CONSORT-AI extension	*Nature Medicine*		
Moons et al,^74^ 2012	Risk	Risk prediction models: I. Development, internal validation, and assessing the incremental value of a new (bio)marker	*Heart*	1320	41
Moons et al,^24^ 2012		Risk prediction models: II. External validation, model updating, and impact assessment	*Heart*		
Chan et al,^75^ 2013	SPIRIT-AI	SPIRIT 2013 Statement: Defining Standard Protocol Items for Clinical Trials	*Annals of Internal Medicine*	2952	75
Chan et al,^79^ 2013		SPIRIT 2013 explanation and elaboration: guidance for protocols of clinical trials	*BMJ*		
Rivera et al,^33^ 2020		Guidelines for clinical trial protocols for interventions involving artificial intelligence: the SPIRIT-AI extension	*BMJ*		
Steyerberg and Vergouwe,^27^ 2014	ABCD	Toward better clinical prediction models: seven steps for development and an ABCD for validation	*European Heart Journal*	709	33
Moons et al,^28^ 2014	CHARMS	Critical Appraisal and Data Extraction for Systematic Reviews of Prediction Modeling Studies: The CHARMS Checklist	*PLoS Medicine*	565	63
Collins et al,^59^ 2015	TRIPOD	Transparent Reporting of a multivariable prediction model for Individual Prognosis Or Diagnosis (TRIPOD): The TRIPOD Statement	*British Journal of Surgery*	3031	86
Moons et al,^80^ 2015		Transparent Reporting of a multivariable prediction model for Individual Prognosis Or Diagnosis (TRIPOD): Explanation and Elaboration	*Annals of Internal Medicine*		
Cohen et al,^76^ 2016	STARD	STARD 2015 guidelines for reporting diagnostic accuracy studies: explanation and elaboration	*BMJ Open*	711	55
Luo et al,^57^ 2016	Guidelines	Guidelines for Developing and Reporting Machine Learning Predictive Models in Biomedical Research: A Multidisciplinary View	*Journal of Medical Internet Research*	244	49
Breck et al,^25^ 2017	ML test score	The ML Test Score: A Rubric for ML Production Readiness and Technical Debt Reduction	*Proceedings of the 2017 IEEE International Conference on Big Data*	68	34
Wolff et al,^77^ 2019	PROBAST	PROBAST: A Tool to Assess the Risk of Bias and Applicability of Prediction Model Studies	*Annals of Internal Medicine*	284	55
Moons et al,^26^ 2019		PROBAST: A Tool to Assess Risk of Bias and Applicability of Prediction Model Studies: Explanation and Elaboration	*Annals of Internal Medicine*		
Mitchell et al,^29^ 2019	Model Cards	Model Cards for Model Reporting	*Proceedings of the Conference on Fairness, Accountability, and Transparency*	311	49
Sendak et al,^31^ 2020	Model facts labels	Presenting machine learning model information to clinical end users with model facts labels	*NPJ Digital Medicine*	14	37
Hernandez-Boussard et al,^30^ 2020	MINIMAR	MINIMAR (MINimum Information for Medical AI Reporting): developing reporting standards for artificial intelligence in health care	*Journal of the American Medical Information Association*	18	28
Norgeot et al,^58^ 2020	MI-CLAIM checklist	Minimum information about clinical artificial intelligence modeling: the MI-CLAIM checklist	*Nature Medicine*	24	40
Silcox et al,^34^ 2020	Trust and value checklist	AI-Enabled Clinical Decision Support Software: A “Trust and Value Checklist” for Clinicians	*NEJM Catalyst*	2	26

Abbreviations: ABCD, alpha calibration-in-the-large, beta calibration slope, C statistic, decision-curve analysis; AI, artificial intelligence; CHARMS, Checklist for Critical Appraisal and Data Extraction for Systematic Reviews of Prediction Modelling Studies; CONSORT, Consolidated Standards of Reporting Trials; MI-CLAIM, Minimum Information About Clinical Artificial Intelligence Modeling; ML, machine learning; PROBAST, Prediction Model Risk of Bias Assessment Tool; SPIRIT, Standard Protocol Items: Recommendations for Interventional Trials; STARD, Standards for Reporting of Diagnostic Accuracy.

^a^ We included the explanation and elaboration papers for CONSORT, SPIRIT, TRIPOD, and PROBAST. For CONSORT and SPIRIT, we also included the AI-specific extensions. We grouped risk prediction models II with the risk prediction models I.

^b^ Sums the citations for each report, excluding the explanation and elaboration papers as of May 2021.

^c^ Indicates the number of deduplicated items sourced from that guideline.

These model reporting guidelines were published in computer science publications (*Proceedings of the Conference on Fairness, Accountability, and Transparency*^29^ and *Proceedings of the 2017 IEEE International Conference on Big Data*^25^), biomedical informatics journals *(Journal of the American Medical Informatics Association*,^30^ *NPJ Digital Medicine*,^31^ and *Journal of Medical Internet Research*^57^), and clinical journals (*Annals of Internal Medicine*,^26,75,77,80^ *BMJ*,^33,79^ *BMJ Open*,^76^ *Nature Medicine*,^32,58^ *Heart*,^24,74^ *European Heart Journal*,^27^ *PLOS Medicine*,^28^ *NEJM Catalyst,*^34^ *Journal of Clinical Epidemiology,*^78^ *International Journal of Surgery*,^35^ and *British Journal of Surgery*^59^). Four guidelines published between 2010 and 2015 have been cited by other articles more than 1000 times, whereas 4 guidelines were published after 2019 and have been cited fewer than 50 times to date.

Of the 15 reporting guidelines, 11 had examples of how to complete their requested items.^25,26,27,29,30,31,38,74,78,79,80^ However, only 5 showed a full example completing all items for a single model,^27,29,30,31,74^ and only 1 of those models had actually been deployed at a health system.^31,81^

After deduplication, 220 distinct items were requested across the reporting guidelines (eAppendix in the Supplement). A cross-tabulation of the 220 items against the 15 reporting guidelines is provided in eTable 2 in the Supplement. For example, the Transparent Reporting of a Multivariable Prediction Model for Individual Prognosis or Diagnosis (TRIPOD) reporting guideline has more items requesting details on preprocessing,^59^ whereas the Minimum Information About Clinical Artificial Intelligence Modeling (MI-CLAIM) has more items requesting details for model examinations.^58^

Table 2 summarizes the model reporting guidelines in terms of the number of items that map to each stage in the creation and evaluation of a machine learning model (Figure 4 in Jung et al^7^). For example, Model Cards^29^ contributes the most items to fairness in model development (n = 29), whereas model facts labels (n = 10)^31^ or Consolidated Standards of Reporting Trials (CONSORT)-AI (n = 10)^32^ contribute the most items to use case assessment.

**Table 2.** Model Reporting Guidelines With Their Items Mapped Onto Different Stages in the Creation and Evaluation of a Machine Learning Model to Guide Care

Model reporting guideline	No. of items that map to each stage^a^									
	Use case assessment	Model			Practical feasibility	Utility assessment	Deployment design	Execution of workflow	Model monitoring	Prospective evaluation
		Formulation	Development	Development: fairness						
Model cards	8	5	29	9	1	0	0	0	0	0
Model facts labels	10	7	9	0	1	1	0	0	2	1
Guidelines	7	6	31	1	0	1	0	0	1	0
MI-CLAIM	4	3	29	3	0	1	0	0	0	1
MINIMAR	4	4	18	5	0	0	0	0	0	0
TRIPOD	7	9	53	1	0	3	0	0	3	2
CONSORT-AI	10	3	23	6	1	0	0	0	2	19
SPIRIT-AI	9	3	17	1	2	0	0	0	2	18
Trust and value checklist	4	0	9	0	2	1	0	0	4	2
ML test score	0	0	12	4	1	0	0	2	17	0
Risk	2	4	24	0	0	1	0	0	2	6
STARD	8	2	37	6	0	1	0	0	0	0
ABCD	1	3	27	0	0	1	0	0	0	0
CHARMS	5	9	42	1	2	0	0	0	1	4
PROBAST	4	6	41	0	1	1	0	0	1	0
Total	14	14	104	10	5	4	0	2	19	25

Abbreviations: ABCD, alpha calibration-in-the-large, beta calibration slope, C statistic, decision-curve analysis; AI, artificial intelligence; CHARMS, Checklist for Critical Appraisal and Data Extraction for Systematic Reviews of Prediction Modelling Studies; CONSORT, Consolidated Standards of Reporting Trials; MI-CLAIM, Minimum Information About Clinical Artificial Intelligence Modeling; MINIMAR, Minimum Information for Medical AI Reporting; ML, machine learning; PROBAST, Prediction Model Risk of Bias Assessment Tool; SPIRIT, Standard Protocol Items: Recommendations for Interventional Trials; STARD, Standards for Reporting of Diagnostic Accuracy; TRIPOD, Transparent Reporting of a Multivariable Prediction Model for Individual Prognosis or Diagnosis.

^a^ Stages are listed in Figure 4 of Jung et al.^7^ Each cell contains the number of items contributed by the relevant model reporting guideline toward a given stage of the workflow (columns).

Table 3 lists the items requested by at least 10 of the 15 reporting guidelines. The most commonly requested items relate to tasks, such as preprocessing, handling missing data, model performance including handling of uncertainty (eg, CIs, statistical significance) or AUROC, and internal validation. A total of 28 distinct performance metrics were requested (eTable 3 in the Supplement), including AUROC, sensitivity, positive predictive value, and calibration plot.

**Table 3.** Commonly Requested Items Across Reporting Guidelines

Item description^a^	No. of reporting guidelines requesting the item	Task^b^	Stage^c^	Reporting rate, %^d^
Provide any description of the data set (eg, training or study) in question	12	Data composition	Model development	100
Define the output or outcome produced by the model	10	Data composition: output	Model formulation	100
Define the specific local area, environment, or setting of training data and model deployment	10	Study design and/or population	Use case	100
Describe how data were preprocessed (eg, data cleaning, predictor transformation, outlier removal, predictor coding)	10	Preprocessing and data cleaning	Model development	100
Desribe how missing data were handled	10	Preprocessing and data cleaning	Model development	50
Describe parameters used to train and select models, including constraints and penalties added as loss terms (eg, shrinkage penalties)	10	Model building	Model development	58
Provide CIs, statistical significance, or some other handling of uncertainty and variability in model performance metrics	10	Model performance and comparison	Model development	0
Clarify what type of validation was performed, whether internal or external	11	Validation	Model development	100
Describe internal validation strategy to account for model optimism (eg, cross-validation, bootstrapping, data splitting)	11	Validation	Model development	100
Describe performance measures	13	Metrics	Model development	100
AUROC (C index)	11	Metrics: discrimination	Model development	100
Describe how the ML model should be used in clinical context	11	Intended use	Use case	100

Abbreviations: AUROC, area under the receiver operating characteristic curve; ML, machine learning.

^a^ Lists all items requested by at least 10 model reporting guidelines.

^b^ Indicates the item’s related task.

^c^ Indicates stage of clinical predictive model development.^7^

^d^ Indicates the percentage of the model briefs that reported the information requested in the item, where the denominator is the number of model briefs for which the item was applicable.

Finally, 77 items were requested by just 1 reporting guideline (eTable 4 in the Supplement). Twelve of the items were model performance metrics such as the *F* score. The ML Test Score had 20 unique items related to model deployment and monitoring, such as the model updating process. CONSORT-AI and Standard Protocol Items: Recommendations for Interventional Trials (SPIRIT)-AI had a combined 21 clinical trial–specific items, which mostly did not apply to Epic System Corporation’s model briefs.

### Reporting of Items by Model Briefs

Interrater agreement on assessments of item reporting was 76% (for all pairs of reviewers, and every item of a given model brief). Of 220 items, 176 (80%) were applicable to at least 1 model brief. Of these, 119 items (68%) were reported by at least 1 model brief. Model briefs reported a median of 39% (IQR, 37%-43%; range, 31%-47%) of applicable items (eTable 5 in the Supplement). After excluding items corresponding to performance metrics—to avoid penalizing model briefs for not reporting multiple, nearly redundant performance metrics—the median completion rate for applicable items was 43% (IQR, 41%-48%; range, 33%-52%). Overall, items had a median reporting rate across model briefs of 25% (IQR, 0-83%; range, 0-100%).

Forty items were reported by more than 90% of the model briefs (eTable 6 in the Supplement). These commonly reported items include information about model development and formulation, specifically the training data set, preprocessing, model type, internal validation, and performance metrics. These items include 9 of the 12 most commonly requested items by the reporting guidelines (Table 3). All 12 model briefs reported the following use case–related items: how the model is to be used in clinical care, who will use the model, ways the model could impact clinical care, and rationale for use.

Seventy-five items were reported by fewer than 10% of the model briefs (eTable 7 in the Supplement). These items included missing data statistics, blinding of predictor and/or outcome assessors, variability of performance measures (eg, CIs), reporting of model coefficients or most predictive features, model examinations including performance errors and intersectional subgroup analyses, user-facing materials and warnings on when to stop use of model, and monitoring of input data and model predictions. In addition, of 28 distinct performance metrics requested, only AUROC (100%), positive predictive value (67%), and sensitivity (42%) were reported by more than one-fifth of the model briefs (eTable 3 in the Supplement).

### Adherence to Entire Reporting Guidelines by Model Briefs

Table 4 shows the adherence rates to individual reporting guidelines, which is the model briefs’ mean completion rate of items requested by each reporting guideline. Model reporting guidelines had a median adherence rate of 53% (IQR, 50%-63%; range, 18%-74%). The ML Test Score had the lowest median adherence rate (18% [IQR, 11%-25%]), whereas Model Facts Labels had the highest (74% [IQR, 71%-80%]). After excluding items corresponding to performance metrics as before, the median adherence rates remained similar, at 57% (IQR, 50%-70%; range, 16%-73%).

**Table 4.** Adherence Rates to Entire Reporting Guidelines Across Model Briefs

Model reporting guideline	Epic Systems Corporation model briefs, %												Mean (IQR), %	No. of applicable items, mean (IQR)
	Deterioration index	Early detection of sepsis	Risk		Pediatric risk of hospital admission or ED visit	Risk of hospital admission or ED visit	Inpatient risk of falls	Projected block utilization	Remaining length of stay	Risk				
			Unplanned readmission	Patient no-show						Admission for heart failure	Hospital admission or ED visit for asthma	Hypertension		
Model cards	66	47	63	51	40	69	51	45	50	47	41	57	52 (46-58)	48.6 (48.0-49.0)
Model facts labels	77	71	80	89	71	80	71	71	82	60	63	71	74 (71-80)	34.8 (35.0-35.0)
Guidelines	64	66	66	66	57	74	62	49	70	64	64	66	64 (63-66)	46.9 (47.0-47.0)
MI-CLAIM	55	58	63	58	47	68	53	34	51	53	45	58	54 (50-58)	37.9 (38.0-38.0)
MINIMAR	71	71	79	61	68	86	71	46	67	75	61	82	70 (65-76)	27.9 (28.0-28.0)
TRIPOD	63	63	61	48	42	61	47	36	57	48	44	51	52 (46-61)	75.4 (74.8-76.0)
CONSORT-AI	63	43	63	60	33	67	53	47	47	49	42	51	52 (46-61)	42.4 (42.0-43.0)
SPIRIT-AI	61	55	54	54	38	61	44	49	51	41	39	46	49 (43-54)	40.4 (40.0-41.0)
Trust and value checklist	46	33	39	50	29	42	38	46	50	25	33	46	40 (33-46)	23.9 (24.0-24.0)
ML test score	27	15	33	24	9	33	15	6	18	12	9	15	18 (11-25)	32.9 (33.0-33.0)
Risk	64	65	63	53	50	68	53	48	61	56	56	56	58 (53-63)	33.6 (33.0-34.0)
STARD	54	45	50	40	29	52	52	39	35	40	40	52	44 (39-52)	48.7 (48.0-49.0)
ABCD	65	65	48	55	61	68	52	39	60	65	61	61	58 (54-65)	30.9 (48.0-49.0)
CHARMS	78	70	68	65	56	75	66	47	73	65	63	64	66 (64-71)	54.9 (54.0-55.0)
PROBAST	69	71	67	62	53	68	58	46	63	60	58	60	61 (58-67)	52.1 (51.8-52.5)

Abbreviations: ABCD, alpha calibration-in-the-large, beta calibration slope, C statistic, decision-curve analysis; AI, artificial intelligence; CHARMS, Checklist for Critical Appraisal and Data Extraction for Systematic Reviews of Prediction Modelling Studies; CONSORT, Consolidated Standards of Reporting Trials; ED, emergency department; MI-CLAIM, Minimum Information About Clinical Artificial Intelligence Modeling; MINIMAR, Minimum Information for Medical AI Reporting; ML, machine learning; PROBAST, Prediction Model Risk of Bias Assessment Tool; SPIRIT, Standard Protocol Items: Recommendations for Interventional Trials; STARD, Standards for Reporting of Diagnostic Accuracy; TRIPOD, Transparent Reporting of a Multivariable Prediction Model for Individual Prognosis or Diagnosis.

### Requested But Less Reported Items

We identified 29 items that were requested by at least 4 of 15 reporting guidelines but were reported by 50% or fewer of model briefs (Table 5). Many of these less-reported items are related to measures of reliability. These include performance of an external validation (33%) and CIs or statistical significance in model performance metrics (0). There was also low reporting of statistics on the amount of missing data (8%) and how missing data were handled (50%). In addition, there was less reporting on items related to fairness (eg, data set representativeness and performance across subgroups). These include summary statistics of key characteristics of the training data set (reporting rate, 50%) or disaggregating performance by a subgroup (33%). Demographic factors such as age (50%), sex (33%), and other relevant factors (50%) lacked both summary statistics and disaggregated performance. Furthermore, there was low reporting of guidance on how to deploy the machine learning model into a clinical workflow (33%), what user-facing materials there will be with the model (0), and how models are updated (42%). Last, some items related to transparency were provided less often, including model coefficients (8%), who funded the study (which might be relevant for conflict of interest purposes) (0), and how to access the data set (0).

**Table 5.** Requested but Less-Reported Items^a^

Item description	Reporting rate, %	No. of model briefs		No. of model reporting guidelines requesting the item
		Applicable	Reporting	
Specify who funded or supported the study and clarify any conflicts of interest	0	10	0	4
Provide information on how to access the data used	0	12	0	4
Provide statistics on the amount of missing data	8	12	1	5
Given the problem context, describe what factors or subgroups would be helpful to perform a subanalysis of model performance evaluation (eg, demographics, environment, lighting); these factors do not have to be available in the data	42	12	5	5
Provide summary statistics of key demographics, characteristics, or other factors for the data set in question	50	12	6	6
Discuss age as an important demographic factor to report summary statistics on or disaggregate performance by	50	12	6	4
Discuss sex as an important demographic factor to report summary statistics on or disaggregate performance by	33	12	4	4
Discuss other factors for the prediction problem to report summary statistics on or disaggregate performance by (eg, sex, sexual orientation, Fitzpatrick skin type, socioeconomic status, geographic location, presenting symptoms, clinical signs, laboratory values, and other diagnoses)	50	12	6	4
Provide flowchart describing how participants were interacted with, assigned, and followed up in the study (especially in clinical trials)	0	12	0	5
Describe the annotation process of the input data, including who annotated the input data, what instructions they were given, and what expertise was needed	18	11	2	4
Describe blinding of data collectors and predictor assessors to outcomes, if done	0.0	9	0	4
Describe the annotation process of the output data, including who annotated the output data, what instructions they were given, and what expertise was needed	27	11	3	7
Describe blinding of outcome assessors to predictors of the model, if done	0	9	0	7
Describe how missing data were handled	50	12	6	10
Indicate whether feature selection involved computing univariate associations between input features and outcomes (not recommended)	18	11	2	4
Provide CIs, statistical significance, or some other handling of uncertainty and variability in model performance metrics	0	12	0	10
Provide sufficient information to enable reproducibility or replication	0	12	0	7
Report model coefficients (regression) or saliency map	8	12	1	7
Disaggregate performance by subgroup or other important data slice	33	12	4	8
Describe external validation strategy and evaluation data set (eg, what external data set was used), ways it may differ from the training set (eg, geography, time), and why the data set was chosen	33	12	4	9
Provide calibration plot	0	12	0	6
Provide negative predictive value	17	12	2	6
Provide sensitivity, ideally at a predefined probability threshold	42	12	5	9
Provide specificity, ideally at a predefined probability threshold	8	12	1	8
Net reclassification improvement	0	12	0	5
Specify directions, explanations, and other user-facing materials that will be included in the model	0	12	0	9
Guidance on how to deploy the machine learning model into clinical workflows	33	12	4	7
Indicate which version of the model is being discussed	45	11	5	6
Describe how models are updated or locally tuned	42	12	5	8

^a^ All items requested by 4 or more model reporting guidelines but reported by no more than 50% of applicable model briefs are listed.

## Discussion

The research community has published many model reporting guidelines with the goal of improving the transparency of prediction models for informed decisions about which models to deploy. However, among 15 reporting guidelines, 220 items are collectively requested, which is both burdensome for model developers to report in their entirety and overwhelming for an end user. We found that documentation examined consistently reported the most requested items from this collective set, but overall a median of 39% of applicable items could be reported. This discrepancy underscores the urgent need to identify items that are both feasible to report in practice and necessary to support a decision to deploy a given clinical prediction model. Adhering to a single model reporting guideline may be insufficient because no single guideline is fully comprehensive, and some items may be familiar only to certain model development communities or have only recently been recognized as relevant. Our approach identified patterns in terms of frequently requested items across guidelines and corresponding gaps in reporting that inform the following suggestions on reporting model information for both the research community and model developers.

For model developers, we suggest prioritizing reporting of the most commonly requested items (Table 3). Model briefs were excellent at reporting these: 9 of the 12 most commonly requested items had 100% reporting rates. These included information on model development and use, such as the outcome definition, and how the model is intended to be used. These commonly requested items—which tend to be about model performance—are not always the most important for making a decision for deployment and do not inform us whether a model will be useful.^7,82^

These 12 commonly requested items are only a subset of what guidelines consider important to report. Therefore, we suggest additional focus on items that were requested but were not often reported (Table 5), such as items related to reliability: external validation, data missingness, and monitoring. Specific example items include external validation strategy, uncertainty measures such as CIs, calibration plots, performance comparison against a baseline, missing data statistics and strategy of missingness handling, how models are updated and tuned, and methods for monitoring input data or regressions in prediction quality in newer data. We further suggest reporting items related to fairness (in this interpretation, referring to data set representativeness and model performance for subgroups) and transparency, which were also often requested but not reported (Table 5). For fairness, model documentation should report summary statistics or disaggregated performance by sex, age, race and ethnicity, and other relevant attributes, as well as the results of subgroup and intersectional analyses. We acknowledge this is a limited view of fairness (which is becoming better defined by a dedicated field of scholarship)^83^ and that items must be contextualized depending on how the model is used and how the data are collected. For example, biased outcome measurement would not be surfaced by subgroup analyses of performance.^6^ For transparency, we suggest reporting model coefficients, model reproducibility, how to access the data set, and who funded the study, which might be relevant for conflict of interest purposes. That these items were rarely reported in the documentation may be unsurprising given that companies have to protect intellectual property such as model architecture details and coefficients, although there is increasing pressure to demonstrate external validation.^19,84^

We suggest that the research community directly engage model developers and information technologists to ensure that published recommendations are feasible to follow and relevant for deployment decisions. As a positive development, dialogue with Epic Systems Corporation’s data science team based on the article’s preprint led to updates to model briefs to include CIs for performance metrics, information about the missing data imputation strategy used, and additional details about algorithm types including, where applicable, parameters used in grid search and type of penalization.^47,85,86^ Such interactions, but occurring at a larger scale, are necessary to bridge the implementation gap by ensuring developers are providing the most relevant and necessary information about their models.

Because many model reporting guidelines^29,30,31,34,58^ aim to support model developers and users, we think recommendations are applicable to model briefs and there is a need for an open forum for bidirection conversation. In eTable 2 and the eAppendix in the Supplement, we group the 220 items by task to enable conversation about which additional items are relevant. Finally, we suggest that deployment teams use items as checklists for ensuring quality in model development, usefulness, workflow capacity, and reliability monitoring^25^ and that teams review items at project initiation time.^87^

### Limitations

This study has several key limitations. First, we analyzed model documentation from only a single vendor, Epic Systems Corporation. Documentation for models at other vendors, such as the Cerner model for patient volume,^88^ could also be analyzed through this framework. Also, to respect copyright, we were not able to release the sections of the model brief that our reviewers used to justify when an item was reported. In addition, although reviewers worked independently, future work could improve on our process for adjudication. Interrater agreement of 76% suggests opportunities to improve reporting. Items that lacked consensus across all model briefs (eTable 8 in the Supplement) often required subjective judgments, such as whether certain items applied if the model brief was not a research study (eg, “Describe how participants were enrolled or recruited into the data” or “Describe the design of the study that was used to collect the data”). Others involved judgments about what reporting was sufficient, such as “Discuss any limitations and caveats of the study.” Methods to assess reporting adherence could be made more consistent and specific through more granular rubrics for third-party reviewers (eg, “partially provided” or “don’t know” categories).

Our findings should be interpreted with caution because our deduplication process may mask certain differences among guidelines (eg, some guidelines provide explicit instructions and examples, whereas others merely call for reporting). We also caution against overinterpreting the completion rate across all items, because items are not exchangeable entities. Two items such as “missing data statistics” and “sensitivity” provide different information, so we recommend considering the completion of individual items when possible. In addition, we were unable to directly assess which items are useful for making deployment decisions, so not every item may be equally important to report. Last, to provide an upper bound on the quality of reporting, reviewers were instructed that in situations for which they were uncertain how to score a particular item, to err on the side of affirming that the item was addressed. For example, we gave credit for “describe how models were tested in a new setting before deployment” for statements that simply stated to contact a support representative to validate the model.

## Conclusions

Model reporting guidelines have been developed to ensure that deployed clinical predictive models are reliable and fair. Although many have been published, to our knowledge they have not been gathered and analyzed in aggregate. In this study, we compile reportable items from 15 reporting guidelines and found that guidelines collectively request 220 distinct items. Such a wide breadth of items collectively poses a large reporting burden for model developers. To provide a snapshot of reporting quality for deployed models, we examined the 12 most adopted models from a single widely used health vendor. We found that the documentation reports the most commonly requested items. However, the documentation could provide more information on reliability, transparency, and fairness. Direct engagement with the vendor led to improvements in their documentation for future users. Overall, there is a need for better prioritization of items to report for predictive models in health care and thereby aid informed decisions about which models to deploy.

## Notes

Supplement. **eMethods.** Model Reporting Search and Model Brief Background **eResults.** Adjudication **eTable 1.** Summary of Epic Model Briefs Reviewed **eTable 2.** Model Reporting Guidelines by Tasks **eTable 3.** Requested Metrics **eTable 4.** Uniquely Requested Items **eTable 5.** Epic Model Brief Completion Rates **eTable 6.** Commonly Reported Items **eTable 7.** Rarely Reported Items **eTable 8.** Low Consensus Items **eAppendix.** Code for Methods, Grading of Model Briefs, Adjudication, and Analysis
eReferences

## References

[1] Rajkomar A, Oren E, Chen K, . Scalable and accurate deep learning with electronic health records. NPJ Digit Med. 2018;1:18. doi:10.1038/s41746-018-0029-1 31304302 PMC6550175

[2] Obermeyer Z, Powers B, Vogeli C, Mullainathan S. Dissecting racial bias in an algorithm used to manage the health of populations. Science. 2019;366(6464):447-453. doi:10.1126/science.aax2342 31649194

[3] Saria S, Butte A, Sheikh A. Better medicine through machine learning: what’s real, and what’s artificial? PLoS Med. 2018;15(12):e1002721. doi:10.1371/journal.pmed.1002721 30596635 PMC6312199

[4] Emanuel EJ, Wachter RM. Artificial intelligence in health care: will the value match the hype? JAMA. 2019;321(23):2281-2282. doi:10.1001/jama.2019.4914 31107500

[5] Topol EJ. High-performance medicine: the convergence of human and artificial intelligence. Nat Med. 2019;25(1):44-56. doi:10.1038/s41591-018-0300-7 30617339

[6] Obermeyer Z, Weinstein JN. Adoption of artificial intelligence and machine learning is increasing, but irrational exuberance remains. NEJM Catalyst. Published online December 10, 2019. doi:10.1056/CAT.19.1090

[7] Jung K, Kashyap S, Avati A, . A framework for making predictive models useful in practice. J Am Med Inform Assoc. 2021;28(6):1149-1158. doi:10.1093/jamia/ocaa318 33355350 PMC8200271

[8] Beam AL, Manrai AK, Ghassemi M. Challenges to the reproducibility of machine learning models in health care. JAMA. 2020;323(4):305-306. doi:10.1001/jama.2019.20866 31904799 PMC7335677

[9] Matheny ME, Whicher D, Thadaney Israni S. Artificial intelligence in health care: a report from the National Academy of Medicine. JAMA. 2020;323(6):509-510. doi:10.1001/jama.2019.21579 31845963

[10] Gianfrancesco MA, Tamang S, Yazdany J, Schmajuk G. Potential biases in machine learning algorithms using electronic health record data. JAMA Intern Med. 2018;178(11):1544-1547. doi:10.1001/jamainternmed.2018.3763 30128552 PMC6347576

[11] Paulus JK, Kent DM. Predictably unequal: understanding and addressing concerns that algorithmic clinical prediction may increase health disparities. NPJ Digit Med. 2020;3:99. doi:10.1038/s41746-020-0304-9 32821854 PMC7393367

[12] Rajkomar A, Hardt M, Howell MD, Corrado G, Chin MH. Ensuring fairness in machine learning to advance health equity. Ann Intern Med. 2018;169(12):866-872. doi:10.7326/M18-1990 30508424 PMC6594166

[13] Parikh RB, Teeple S, Navathe AS. Addressing bias in artificial intelligence in health care. JAMA. 2019;322(24):2377-2378. doi:10.1001/jama.2019.18058 31755905

[14] Coley RY, Johnson E, Simon GE, Cruz M, Shortreed SM. Racial/ethnic disparities in the performance of prediction models for death by suicide after mental health visits. JAMA Psychiatry. 2021;78(7):726-734. doi:10.1001/jamapsychiatry.2021.0493 33909019 PMC8082428

[15] Park Y, Hu J, Singh M, . Comparison of methods to reduce bias from clinical prediction models of postpartum depression. JAMA Netw Open. 2021;4(4):e213909. doi:10.1001/jamanetworkopen.2021.3909 33856478 PMC8050742

[16] Seyyed-Kalantari L, Liu G, McDermott M, Chen IY, Ghassemi M. CheXclusion: fairness gaps in deep chest x-ray classifiers. Pac Symp Biocomput. 2021;26:232-243. 33691020

[17] Barda N, Yona G, Rothblum GN, . Addressing bias in prediction models by improving subpopulation calibration. J Am Med Inform Assoc. 2021;28(3):549-558. doi:10.1093/jamia/ocaa283 33236066 PMC7936516

[18] Pfohl SR, Foryciarz A, Shah NH. An empirical characterization of fair machine learning for clinical risk prediction. J Biomed Inform. 2021;113:103621. doi:10.1016/j.jbi.2020.103621 33220494 PMC7871979

[19] Khetpal V, Shah N. How a largely untested AI algorithm crept into hundreds of hospitals. May 28, 2021. Accessed June 25, 2021. https://www.fastcompany.com/90641343/epic-deterioration-index-algorithm-pandemic-concerns

[20] Wu E, Wu K, Daneshjou R, Ouyang D, Ho DE, Zou J. How medical AI devices are evaluated: limitations and recommendations from an analysis of FDA approvals. Nat Med. 2021;27(4):582-584. doi:10.1038/s41591-021-01312-x 33820998

[21] Lecher C. What happens when an algorithm cuts your health care. The Verge. March 21, 2018. Accessed June 2, 2021. https://www.theverge.com/2018/3/21/17144260/healthcare-medicaid-algorithm-arkansas-cerebral-palsy

[22] Reuter E. Popular sepsis prediction model works “substantially worse” than claimed, researchers find. MedCity News. June 23, 2021. Accessed June 28, 2021. https://medcitynews.com/2021/06/popular-sepsis-prediction-model-works-substantially-worse-than-claimed-researchers-find/

[23] Wong A, Otles E, Donnelly JP, . External validation of a widely implemented proprietary sepsis prediction model in hospitalized patients. JAMA Intern Med. 2021;181(8):1065-1070. doi:10.1001/jamainternmed.2021.2626 34152373 PMC8218233

[24] Moons KGM, Kengne AP, Grobbee DE, . Risk prediction models, II: external validation, model updating, and impact assessment. Heart. 2012;98(9):691-698. doi:10.1136/heartjnl-2011-301247 22397946

[25] Breck E, Cai S, Nielsen E, Salib M, Sculley D. The ML Test Score: a rubric for ML production readiness and technical debt reduction. *Proceedings of the 2017 IEEE International Conference on Big Data*. December 11-14, 2017:1123-1132. doi:10.1109/BigData.2017.8258038

[26] Moons KGM, Wolff RF, Riley RD, . PROBAST: a tool to assess risk of bias and applicability of prediction model studies: explanation and elaboration. Ann Intern Med. 2019;170(1):W1-W33. doi:10.7326/M18-1377 30596876

[27] Steyerberg EW, Vergouwe Y. Towards better clinical prediction models: seven steps for development and an ABCD for validation. Eur Heart J. 2014;35(29):1925-1931. doi:10.1093/eurheartj/ehu207 24898551 PMC4155437

[28] Moons KGM, de Groot JAH, Bouwmeester W, . Critical appraisal and data extraction for systematic reviews of prediction modelling studies: the CHARMS checklist. PLoS Med. 2014;11(10):e1001744. doi:10.1371/journal.pmed.1001744 25314315 PMC4196729

[29] Mitchell M, Wu S, Zaldivar A, . Model Cards for model reporting. In: *Proceedings of the Conference on Fairness, Accountability, and Transparency*. Association for Computing Machinery; January 29, 2019.

[30] Hernandez-Boussard T, Bozkurt S, Ioannidis JPA, Shah NH. MINIMAR (Minimum Information for Medical AI Reporting): developing reporting standards for artificial intelligence in health care. J Am Med Inform Assoc. 2020;27(12):2011-2015. doi:10.1093/jamia/ocaa088 32594179 PMC7727333

[31] Sendak MP, Gao M, Brajer N, Balu S. Presenting machine learning model information to clinical end users with model facts labels. NPJ Digit Med. 2020;3:41. doi:10.1038/s41746-020-0253-3 32219182 PMC7090057

[32] Liu X, Cruz Rivera S, Moher D, Calvert MJ, Denniston AK; SPIRIT-AI and CONSORT-AI Working Group. Reporting guidelines for clinical trial reports for interventions involving artificial intelligence: the CONSORT-AI extension. Nat Med. 2020;26(9):1364-1374. doi:10.1038/s41591-020-1034-x 32908283 PMC7598943

[33] Rivera SC, Liu X, Chan AW, Denniston AK, Calvert MJ; SPIRIT-AI and CONSORT-AI Working Group. Guidelines for clinical trial protocols for interventions involving artificial intelligence: the SPIRIT-AI Extension. BMJ. 2020;370:m3210. doi:10.1136/bmj.m3210 32907797 PMC7490785

[34] Silcox C, Dentzer S, Bates DW. AI-enabled clinical decision support software: a “trust and value checklist” for clinicians. NEJM Catalyst. 2020;1(6). doi:10.1056/CAT.20.0212

[35] Schulz KF, Altman DG, Moher D; CONSORT Group. CONSORT 2010 statement: updated guidelines for reporting parallel group randomised trials. Int J Surg. 2011;9(8):672-677. doi:10.1016/j.ijsu.2011.09.004 22019563

[36] Chan AW, Tetzlaff JM, Altman DG, Dickersin K, Moher D. SPIRIT 2013: new guidance for content of clinical trial protocols. Lancet. 2013;381(9861):91-92. doi:10.1016/S0140-6736(12)62160-6 23305999

[37] von Elm E, Altman DG, Egger M, Pocock SJ, Gøtzsche PC, Vandenbroucke JP; STROBE Initiative. Strengthening the Reporting of Observational Studies in Epidemiology (STROBE) statement: guidelines for reporting observational studies. BMJ. 2007;335(7624):806-808. doi:10.1136/bmj.39335.541782.AD 17947786 PMC2034723

[38] Bossuyt PM, Reitsma JB, Bruns DE, ; STARD Group. STARD 2015: an updated list of essential items for reporting diagnostic accuracy studies. BMJ. 2015;351:h5527. doi:10.1136/bmj.h5527 26511519 PMC4623764

[39] Glazer D, Tabak LA. Artificial Intelligence Working Group Update. ACD Working Group on Artificial Intelligence: 119th Meeting of the Advisory Committee to the Director (ACD). December 13, 2019. Accessed June 24, 2021. https://acd.od.nih.gov/documents/presentations/12132019AI.pdf

[40] DECIDE-AI Steering Group. DECIDE-AI: new reporting guidelines to bridge the development-to-implementation gap in clinical artificial intelligence. Nat Med. 2021;27(2):186-187. doi:10.1038/s41591-021-01229-5 33526932

[41] Collins GS, Moons KGM. Reporting of artificial intelligence prediction models. Lancet. 2019;393(10181):1577-1579. doi:10.1016/S0140-6736(19)30037-6 31007185

[42] Sounderajah V, Ashrafian H, Aggarwal R, . Developing specific reporting guidelines for diagnostic accuracy studies assessing AI interventions: the STARD-AI Steering Group. Nat Med. 2020;26(6):807-808. doi:10.1038/s41591-020-0941-1 32514173

[43] Bozkurt S, Cahan EM, Seneviratne MG, . Reporting of demographic data and representativeness in machine learning models using electronic health records. J Am Med Inform Assoc. 2020;27(12):1878-1884. doi:10.1093/jamia/ocaa164 32935131 PMC7727384

[44] Wynants L, Van Calster B, Collins GS, . Prediction models for diagnosis and prognosis of COVID-19: systematic review and critical appraisal. BMJ. 2020;369:m1328. doi:10.1136/bmj.m1328 32265220 PMC7222643

[45] Epic Systems Corporation. Cognitive computing model brief: deterioration index. January 8, 2021. Accessed March 8, 2021. https://galaxy.epic.com/?#Browse/page=1!68!50!3883949

[46] Cognitive Computing model brief: early detection of sepsis. Epic Systems Corp. December 13, 2016. Accessed March 8, 2021. https://galaxy.epic.com/?#Browse/page=1!68!50!3289911

[47] Epic Systems Corporation. Cognitive computing model brief: risk of unplanned readmission (version 2). May 3, 2020. Accessed March 8, 2021. https://galaxy.epic.com/Redirect.aspx?DocumentID=100051822

[48] Epic Systems Corporation. Cognitive computing model brief: risk of patient no-show (version 2). January 29, 2021. Accessed March 8, 2021. https://galaxy.epic.com/Redirect.aspx?DocumentID=100020266

[49] Epic Systems Corporation. Cognitive computing model brief: pediatric hospital admissions and ED visits. March 31, 2018. Accessed March 8, 2021. https://galaxy.epic.com/Redirect.aspx?DocumentID=3763630&Version=Epic%202018

[50] Epic Systems Corporation. Cognitive computing model brief: risk of hospital admission or ED visit (version 2). May 1, 2020. Accessed March 8, 2021. https://galaxy.epic.com/Redirect.aspx?DocumentID=100045918

[51] Epic Systems Corporation. Cognitive computing model brief: inpatient risk of falls. September 2, 2020. Accessed April 13, 2021. https://galaxy.epic.com/?#Browse/page=1!68!50!100014430

[52] Epic Systems Corporation. Cognitive computing model brief: projected block utilization. August 29, 2018. Accessed April 13, 2021. https://galaxy.epic.com/Redirect.aspx?DocumentID=100014389

[53] Epic Systems Corporation. Cognitive computing model brief: remaining length of stay. April 7, 2017. Accessed April 13, 2021. https://galaxy.epic.com/Redirect.aspx?DocumentID=3364171&Version=Epic%202018

[54] Epic Systems Corporation. Cognitive computing model brief: hospital admissions for heart failure. November 1, 2017. Accessed April 13, 2021. https://galaxy.epic.com/Redirect.aspx?DocumentID=3706332&Version=Epic%202018

[55] Epic Systems Corporation. Cognitive computing model brief: hospital admissions and ED visits for asthma. August 29, 2017. Accessed April 13, 2021. https://galaxy.epic.com/Redirect.aspx?DocumentID=3587370

[56] Epic Systems Corporation. Cognitive computing model brief: hypertension. December 13, 2016. Accessed April 13, 2021. https://galaxy.epic.com/?#Browse/page=1!68!50!3479172

[57] Luo W, Phung D, Tran T, . Guidelines for developing and reporting machine learning predictive models in biomedical research: a multidisciplinary view. J Med Internet Res. 2016;18(12):e323. doi:10.2196/jmir.5870 27986644 PMC5238707

[58] Norgeot B, Quer G, Beaulieu-Jones BK, . Minimum Information About Clinical Artificial Intelligence Modeling: the MI-CLAIM checklist. Nat Med. 2020;26(9):1320-1324. doi:10.1038/s41591-020-1041-y 32908275 PMC7538196

[59] Collins GS, Reitsma JB, Altman DG, Moons KGM. Transparent Reporting of a Multivariable Prediction Model for Individual Prognosis or Diagnosis (TRIPOD): the TRIPOD statement. Br J Surg. 2015;102(3):148-158. doi:10.1002/bjs.9736 25627261

[60] Watson J, Hutyra CA, Clancy SM, . Overcoming barriers to the adoption and implementation of predictive modeling and machine learning in clinical care: what can we learn from US academic medical centers? JAMIA Open. 2020;3(2):167-172. doi:10.1093/jamiaopen/ooz046 32734155 PMC7382631

[61] Kent DM, Paulus JK, van Klaveren D, . The Predictive Approaches to Treatment Effect Heterogeneity (PATH) statement. Ann Intern Med. 2020;172(1):35-45. doi:10.7326/M18-3667 31711134 PMC7531587

[62] Yu B, Kumbier K. Veridical data science. Proc Natl Acad Sci U S A. 2020;117(8):3920-3929. doi:10.1073/pnas.1901326117 32054788 PMC7049126

[63] Setting guidelines to report the use of AI in clinical trials. Nat Med. 2020;26(9):1311. doi:10.1038/s41591-020-1069-z 32908276

[64] The Lancet Digital Health. Guiding better design and reporting of AI-intervention trials. Lancet Digit Health. 2020;2(10):e493. doi:10.1016/S2589-7500(20)30223-5 33328046

[65] Corey KM, Helmkamp J, Simons M, . Assessing quality of surgical real-world data from an automated electronic health record pipeline. J Am Coll Surg. 2020;230(3):295-305.e12. doi:10.1016/j.jamcollsurg.2019.12.005 31945461

[66] Veinot TC, Mitchell H, Ancker JS. Good intentions are not enough: how informatics interventions can worsen inequality. J Am Med Inform Assoc. 2018;25(8):1080-1088. doi:10.1093/jamia/ocy052 29788380 PMC7646885

[67] Bender EM, Friedman B. Data statements for natural language processing: toward mitigating system bias and enabling better science. Trans Assoc Comput Linguist. 2018;6:587-604. doi:10.1162/tacl_a_00041

[68] Gebru T, Morgenstern J, Vecchione B, . Datasheets for datasets. arXiv. Preprint posted online March 23, 2018. doi:10.48550/arXiv.1830.09010

[69] Wynants L, Smits LJM, Van Calster B. Demystifying AI in healthcare. BMJ. 2020;370:m3505. doi:10.1136/bmj.m3505 32907834

[70] Eaneff S, Obermeyer Z, Butte AJ. The case for algorithmic stewardship for artificial intelligence and machine learning technologies. JAMA. 2020;324(14):1397-1398. doi:10.1001/jama.2020.9371 32926087

[71] Nagendran M, Chen Y, Lovejoy CA, . Artificial intelligence versus clinicians: systematic review of design, reporting standards, and claims of deep learning studies. BMJ. 2020;368:m689. doi:10.1136/bmj.m689 32213531 PMC7190037

[72] Wiens J, Saria S, Sendak M, . Do no harm: a roadmap for responsible machine learning for health care. Nat Med. 2019;25(9):1337-1340. doi:10.1038/s41591-019-0548-6 31427808

[73] Park Y, Jackson GP, Foreman MA, Gruen D, Hu J, Das AK. Evaluating artificial intelligence in medicine: phases of clinical research. JAMIA Open. 2020;3(3):326-331. doi:10.1093/jamiaopen/ooaa033 33215066 PMC7660958

[74] Moons KGM, Kengne AP, Woodward M, . Risk prediction models, I: development, internal validation, and assessing the incremental value of a new (bio)marker. Heart. 2012;98(9):683-690. doi:10.1136/heartjnl-2011-301246 22397945

[75] Chan A-W, Tetzlaff JM, Altman DG, . SPIRIT 2013 statement: defining standard protocol items for clinical trials. Ann Intern Med. 2013;158(3):200-207. doi:10.7326/0003-4819-158-3-201302050-00583 23295957 PMC5114123

[76] Cohen JF, Korevaar DA, Altman DG, . STARD 2015 guidelines for reporting diagnostic accuracy studies: explanation and elaboration. BMJ Open. 2016;6(11):e012799. doi:10.1136/bmjopen-2016-012799 28137831 PMC5128957

[77] Wolff RF, Moons KGM, Riley RD, ; PROBAST Group†. PROBAST: a tool to assess the risk of bias and applicability of prediction model studies. Ann Intern Med. 2019;170(1):51-58. doi:10.7326/M18-1376 30596875

[78] Moher D, Hopewell S, Schulz KF, ; Consolidated Standards of Reporting Trials Group. CONSORT 2010 explanation and elaboration: updated guidelines for reporting parallel group randomised trials. J Clin Epidemiol. 2010;63(8):e1-e37. doi:10.1016/j.jclinepi.2010.03.004 20346624

[79] Chan AW, Tetzlaff JM, Gøtzsche PC, . SPIRIT 2013 explanation and elaboration: guidance for protocols of clinical trials. BMJ. 2013;346:e7586. doi:10.1136/bmj.e7586 23303884 PMC3541470

[80] Moons KGM, Altman DG, Reitsma JB, . Transparent Reporting of a multivariable prediction model for Individual Prognosis or Diagnosis (TRIPOD): explanation and elaboration. Ann Intern Med. 2015;162(1):W1-W73. doi:10.7326/M14-0698 25560730

[81] Duke Institute for Health Innovation. Sepsis watch: the implementation of a Duke-specific early warning system for sepsis. January 18, 2020. Accessed July 2, 2021. https://dihi.org/project/sepsiswatch/

[82] Shah NH, Milstein A, Bagley SC. Making machine learning models clinically useful. JAMA. 2019;322(14):1351-1352. doi:10.1001/jama.2019.10306 31393527

[83] ACM Conference on Fairness, Accountability, and Transparency (ACM FAccT). June 1, 2021. Accessed July 2, 2021. https://facctconference.org/index.html 10.1145/3593013.3594102 PMC10661580 37990734

[84] Ross C. Epic’s AI algorithms, shielded from scrutiny by a corporate firewall, are delivering inaccurate information on seriously ill patients. STAT. July 26, 2021. Accessed January 31, 2022. https://www.statnews.com/2021/07/26/epic-hospital-algorithms-sepsis-investigation/

[85] Epic Systems Corporation. Cognitive computing model brief. risk of unplanned: readmission (version 2). August 11, 2021. Accessed May 31, 2022. https://galaxy.epic.com/?#Browse/page=1!68!50!100051822

[86] Epic Systems Corporation. Cognitive computing model brief. risk of patient no-show (version 2). February 27, 2022. Accessed May 31, 2022. https://galaxy.epic.com/?#Browse/page=1!68!50!100020266

[87] Jimmerson C. *A3 Problem Solving for Healthcare: A Practical Method for Eliminating Waste*. Productivity Press; 2007.

[88] ORACLE Cerner. From diagnosis to holistic patient care, machine learning is transforming health care. October 21, 2019. Accessed January 24, 2022. https://www.cerner.com/perspectives/machine-learning-is-transforming-health-care

