# Supplementary material for: Assessment of Adherence to Reporting Guidelines by Commonly Used Clinical Prediction Models From a Single Vendor: A Systematic Review
Source: JAMA Netw Open. 2022 Aug 19;5(8):e2227779. doi: 10.1001/jamanetworkopen.2022.27779 (PMC9391954; doi:10.1001/jamanetworkopen.2022.27779)
Supplement: Supplement. — eMethods. Model Reporting Search and Model Brief Background eResults. Adjudication eTable 1. Summary of Epic Model Briefs Reviewed eTable 2. Model Reporting Guidelines by Tasks eTable 3. Requested Metrics eTable 4. Uniquely Requested Items eTable 5. Epic Model Brief Completion Rates eTable 6. Commonly Reported Items eTable 7. Rarely Reported Items eTable 8. Low Consensus Items eAppendix. Code for Methods, Grading of Model Briefs, Adjudication, and Analysis eReferences [file jamanetwopen-e2227779-s001.pdf]

Supplementary Online Content

Lu JH, Callahan A, Patel BS, et al. Assessment of adherence to reporting guidelines by commonly used clinical prediction models from a single vendor: a systematic review. *JAMA Netw Open*. 2022;5(8):e2227779. doi:10.1001/jamanetworkopen.2022.27779

eMethods. Model Reporting Search and Model Brief Background
eResults. Adjudication
eTable 1. Summary of Epic Model Briefs Reviewed
eTable 2. Model Reporting Guidelines by Tasks
eTable 3. Requested Metrics
eTable 4. Uniquely Requested Items
eTable 5. Epic Model Brief Completion Rates
eTable 6. Commonly Reported Items
eTable 7. Rarely Reported Items
eTable 8. Low Consensus Items
eAppendix. Code for Methods, Grading of Model Briefs, Adjudication, and Analysis
eReferences

This supplementary material has been provided by the authors to give readers additional information about their work.

© 2022 Lu JH et al. *JAMA Network Open*.

eMethods. Model Reporting Search and Model Brief Background

In our search for model reporting guideline publications, we included all Explanation and Elaboration documents, AI-specific extensions and multi-part guidelines for papers which had them.

Items had consensus when all four reviewers agreed that an item was reported by the Model Brief, was not reported by the Model Brief, or was determined to be not applicable. For items that did not have consensus across all four reviewers, a designated adjudicator reviewed the items and the corresponding Model Brief content, to independently adjudicate the reviewer responses.

To determine the inter-rater agreement, we calculated the fraction of items that a pair of reviewers agreed were reported, were not reported, or were determined to be not applicable, averaged across all Model Briefs and pairs of the four reviewers.

To standardize nomenclature, we define that an item is "requested" by a reporting guideline if any reportable item from the reporting guideline was merged into that item. We define that an item is "reported" by a Model Brief if we determine that the Model Brief contained the information requested in the item, after adjudication.

An item's reporting rate is the number of Model Briefs that reported the item divided by the number of Model Briefs for which the item was applicable. A Model Brief's completion rate of a given group of items is the number of items reported by the Model Brief divided by the number of items that were applicable to that Model Brief. Finally, the adherence rate to a reporting guideline is the completion rate of items requested by the specific reporting guideline, averaged across all Model Briefs. We calculate median, interquartile range (IQR) and range for items' reporting rates, Model Briefs' completion rates, and reporting guidelines' adherence rates, as appropriate.

In reviewing, reviewers could designate items as "reported", "not reported", "not applicable" and also "wrongly reported." However, for "wrongly reported", this was rare to occur and only one item for one Model Brief was adjudicated to this, so this is not further discussed in the manuscript.

Model Brief Background

We provide a general summary of the 12 models assessed. This is also summarized in eTable 2. We do not provide further detail to avoid violating Epic's copyright policy.

The Deterioration Index model[1] is used for stratifying patients by their risk of clinical deterioration. It takes in features related to demographic information, vital signs, lab results and nursing assessments and outputs an ordinal target relating to whether a patient had an escalation in care or mortality event.

The Early Detection of Sepsis model[2] is used for identifying patients at risk of developing sepsis. It takes in features related to demographics, comorbidities, SIRS criteria, lab results, medication orders, and lines/drains/airways and outputs the probability a patient will become septic or near septic in the near future.

The Risk of Unplanned Readmission (version 2) model[3] is used to identify patients that may have unplanned readmissions to the hospital. It takes in features related to demographics, diagnoses, flowsheet values, labs, medications and healthcare utilization and outputs the probability that a patient will be readmitted to the hospital within 30 days of discharge.

The Risk of Patient No-Show (version 2) model[4] is used to identify appointments that are likely to be a no-show. It takes in features related to demographics, appointment characteristics, and appointment history and outputs the patient's probability to miss their upcoming appointment or cancel it with little notice.

© 2022 Lu JH et al. *JAMA Network Open*.

The Pediatric Hospital Admissions and ED Visits model[5] is used for identifying pediatric patients at-risk of hospital admissions or ED visits. It takes in features related to demographics, diagnoses, medications and healthcare utilization and outputs the probability that a pediatric patient will be hospitalized or visit the ED in the next six months.

The Risk of Hospital Admission or ED Visit (Version 2) model[6] is used for predicting the risk of patients having hospital admission or ED visit within the next year. It takes in features related to demographics, diagnoses, medications, procedures and healthcare utilization and outputs the probability of patients 18 years old and older of visiting the ED or being admitted to the hospital within the next year.

The Inpatient Risk of Falls model[7] is used to provide a risk of falls assessment for patients. It takes in features related to demographics, vital signs, lab results, medications, procedure orders and lines/drains/airways, and outputs an ordinal target related to patients falling and/or receiving interventions to prevent a fall.

The Projected Block Utilization model[8] is used to predict block utilization for surgeons and other services. It takes in features related to block type, day of the week and days prior to surgery and outputs the predicted scheduled utilization on the morning of the surgery.

The Remaining Length of Stay model[9] is used for predicting a patient's remaining length of stay in a hospital. It takes in features related to diagnoses, demographics, flowsheets, labs, medications, orders, certain clinical risk scores and healthcare utilization and outputs a predicted remaining length-of-stay for the patient.

The Hospital Admissions for Heart Failure model[10] is used for predicting which heart failure patients are at high risk of hospitalization. It takes in features related to demographics, diagnoses, medications, healthcare utilization, and labs and outputs the probability that a heart failure patient will be hospitalized.

The Hospital Admissions and ED Visits for Asthma model[11] is used for identifying the asthma patients most at risk of ending up in the ED due to asthma-related conditions. It takes in features related to demographics, social history, diagnoses, medications and immunizations, and healthcare utilization and outputs the probability that an asthma patient will visit the ED or be hospitalized with a diagnosis of asthma in the next 12 months.

The Hypertension model[12] is used for determining which patients have the highest risk of developing hypertension within the next two years. It takes in features related to demographics, diagnoses, family history, vitals, and medications and outputs the probability that a patient will develop hypertension in the next two years.

© 2022 Lu JH et al. *JAMA Network Open*.

eResults. Adjudication

A median of 93 (IQR: 88-95, range: 66-108) items per brief underwent adjudication to resolve disagreement among reviewers. There were 34 items for which reviewers had no consensus across any of the 12 Model Briefs (eTable 8). These items related to data collection, reference standards, and performance metrics, where there was disagreement about applicability.

© 2022 Lu JH et al. *JAMA Network Open*.

eTable 1. Summary of Epic Model Briefs Reviewed

Name of Model Brief	Purpose	Inputs relate to....	Output relates to...	Date of Last Update (at time of study)	Community Adoption Score (out of 3)
Cognitive Computing Model Brief: Deterioration Index	Stratifying patients by their risk of clinical deterioration	Demographic information, vital signs, lab results and nursing assessments	Ordinal target relating to whether a patient had an escalation in care or mortality event	01/08/2021	3
Cognitive Computing Model Brief: Early Detection of Sepsis	Identifying patients at risk of developing sepsis	Demographics, comorbidities, SIRS criteria, lab results, medication orders, and lines/drains/airways	Probability a patient will become septic or near septic in the near future	12/13/2016	3
Cognitive Computing Model Brief: Risk of Unplanned Readmission (Version 2)	Identify patients that may have unplanned readmissions to the hospital	Demographics, diagnoses, flowsheet values, labs, medications and healthcare utilization	Probability that a patient will be readmitted to the hospital within 30 days of discharge	05/03/2020	3
Cognitive Computing Model Brief: Risk of Patient No-Show (Version 2)	Identify appointments that are likely to be a no-show	Demographics, appointment characteristics, and appointment history	Patient's probability to miss their upcoming appointment or cancel it with little notice	01/29/2021	3
Cognitive Computing Model Brief: Pediatric Hospital Admissions and ED Visits	Identify pediatric patients at-risk of hospital admissions or ED visits	Demographics, diagnoses, medications and healthcare utilization	Probability that a pediatric patient will be hospitalized or visit the ED in the next six months	03/13/2018	3
Cognitive Computing Model Brief: Risk of Hospital Admission or ED Visit (Version 2)	Predicting the risk of patients having hospital admission or ED visit within the next year	Demographics, diagnoses, medications, procedures and healthcare utilization	Probability of patients 18 years old and older of visiting the ED or being admitted to the hospital within the next year	05/01/2020	3

© 2022 Lu JH et al. *JAMA Network Open*.

Cognitive Computing Model Brief: Inpatient Risk of Falls	Provide a risk of falls assessment for patients	Demographics, vital signs, lab results, medications, procedure orders and lines/drains/airways	Ordinal target related to patients falling and/or receiving interventions to prevent a fall	09/02/2020	2
Cognitive Computing Model Brief: Projected Block Utilization	Predict block utilization for surgeons and other services	Block type, day of the week and days prior to surgery	Predicted scheduled utilization on the morning of the surgery	08/29/2018	2
Cognitive Computing Model Brief: Remaining Length of Stay	Predicting a patient's remaining length of stay in a hospital	Diagnoses, demographics, flowsheets, labs, medications, orders, certain clinical risk scores and healthcare utilization	Predicted remaining length-of-stay for the patient	04/07/2017	2
Cognitive Computing Model Brief: Hospital Admissions for Heart Failure	Predicting which heart failure patients are at high risk of hospitalization	Demographics, diagnoses, medications, healthcare utilization, and labs	Probability that a heart failure patient will be hospitalized	11/01/2017	2
Cognitive Computing Model Brief: Hospital Admissions and ED Visits for Asthma	Identifying the asthma patients most at risk of ending up in the ED due to asthma-related conditions	Demographics, social history, diagnoses, medications and immunizations, and healthcare utilization	Probability that an asthma patient will visit the ED or be hospitalized with a diagnosis of asthma in the next 12 months	08/29/2017	2
Cognitive Computing Model Brief: Hypertension	Determining which patients have the highest risk of developing hypertension within the next two years	Demographics, diagnoses, family history, vitals, and medications	Probability that a patient will develop hypertension in the next two years	12/13/2016	2

© 2022 Lu JH et al. *JAMA Network Open*.

eTable 2. Model Reporting Guidelines by Tasks

	MODEL REPORTING GUIDELINES															
**TASK**	**Model Cards**	**Model Facts Labels**	**Guide lines**	**MI-CLAIM**	**MINIM AR**	**TRIP OD**	**CONS ORT-AI**	**SPIRI T-AI**	**Trust and Value**	**ML Test Score**	**Risk**	**STARD**	**ABCD**	**CHARMS**	**PROB AST**	**Total Items**
**Overview**	7	8	6	2	1	10	9	**14**	1	0	1	8	2	2	2	28
Overview: Clinical Trial	0	1	0	0	0	1	2	**9**	0	0	0	2	0	0	0	9
**Data Composition**	7	4	8	6	9	10	8	4	1	3	3	10	5	10	11	24
Data Composition: Input	0	1	1	1	1	3	0	0	0	0	1	0	1	3	**4**	5
Data Composition: Factors	**6**	0	0	2	5	1	5	0	0	3	0	5	0	0	0	7
Data Composition: Output	0	2	3	1	1	2	1	1	0	0	1	2	3	**4**	**4**	7
**Study Design/Population**	1	2	2	0	3	**4**	**4**	**4**	0	0	2	2	1	3	**4**	4
**Data Collection & Methods**	6	1	1	0	0	11	4	**12**	6	1	5	8	0	9	10	21
Data Collection & Methods: Input	1	0	0	0	0	**2**	1	1	0	0	0	0	0	1	**2**	2
Data Collection & Methods: Outcome	1	0	0	0	0	4	1	2	1	0	2	4	0	4	**5**	7
Data Collection & Methods: Consent/Privacy for Data	0	0	0	0	0	0	0	**4**	**4**	1	0	0	0	0	0	4

© 2022 Lu JH et al. *JAMA Network Open*.

Evaluation-specific Study Details: Methods	0	0	0	1	0	2	5	**10**	1	0	2	0	0	3	0	13
Evaluation-specific Study Details: Randomization	0	0	0	0	0	0	**4**	3	0	0	1	0	0	0	0	4
Evaluation-specific Study Details: Blinding	0	0	0	0	0	0	3	**5**	0	0	0	0	0	0	0	5
Evaluation-specific Study Details: Outcomes	0	0	0	0	0	0	**4**	1	0	0	1	0	0	0	0	4
Evaluation-specific Study Details: Analysis	0	1	0	0	0	0	**4**	**4**	1	0	2	0	0	1	0	4
**Preprocessing and Data Cleaning**	1	0	3	1	2	4	3	2	0	1	5	1	4	5	**6**	7
**Model Building**	1	0	2	3	2	**5**	1	1	0	3	3	1	3	3	2	8
**Model Summary**	2	1	1	1	1	**3**	1	1	0	0	2	2	1	**3**	1	4
**Model Performance and Comparison**	**1**	0	**1**	**1**	0	**1**	**1**	0	**1**	0	**1**	**1**	**1**	**1**	0	1
**Model Examination**	2	0	5	**9**	0	1	4	3	1	2	1	1	2	3	1	13
**Validation**	3	3	2	2	3	**4**	0	0	3	0	3	0	3	3	3	4
**Metrics**	7	4	8	8	5	**17**	0	0	1	1	5	12	10	10	10	29
Metrics: Discrimination	1	1	1	1	1	**3**	0	0	0	0	1	1	2	1	1	3
Metrics: Goodness-of-Fit	0	0	1	0	0	**3**	0	0	0	0	0	1	1	2	1	6
Metrics: Calibration	0	0	1	0	0	**2**	0	0	0	0	1	0	**2**	1	1	2

© 2022 Lu JH et al. *JAMA Network Open*.

Metrics: Classification	5	2	4	5	3	4	0	0	0	0	0	**9**	2	4	4	12
Metrics: Utility	0	0	0	1	0	**2**	0	0	0	0	0	0	1	0	1	3
Metrics: Compare Two Model Discrimination	0	0	0	0	0	**2**	0	0	0	0	**2**	0	1	1	1	2
**Comparison Against Baseline Model**	0	0	2	**3**	0	2	0	0	0	2	0	1	0	0	0	3
**Intended Use**	3	**6**	3	1	2	3	3	3	3	0	2	3	1	1	1	7
Intended Use: User	1	**2**	1	0	1	1	**2**	**2**	1	0	1	1	1	0	0	2
Intended Use: Warnings	1	**2**	0	0	0	0	0	0	1	0	0	0	0	0	0	3
**Deployment**	2	5	1	1	0	4	5	6	6	**20**	2	1	0	3	2	26
Deployment: Tests: Data	1	0	0	0	0	0	0	1	1	**5**	0	0	0	0	0	6
Deployment: Tests: Infrastructure	0	0	0	0	0	0	0	0	0	**4**	0	0	0	0	0	4
Deployment: Updating	1	2	0	0	0	**4**	3	3	2	**4**	2	0	0	2	1	6
Deployment: Monitoring	0	1	1	0	0	0	0	0	1	**7**	0	0	0	0	0	7
**Ethics**	**3**	1	1	0	0	0	1	1	1	0	0	1	0	0	0	3
**Limitations**	2	1	3	1	0	**4**	**4**	1	0	0	0	3	0	2	1	6
Miscellaneous	**1**	0	0	0	0	**1**	0	0	0	**1**	0	0	0	**1**	**1**	2

Model reporting guidelines (in rows), with their items mapped onto different tasks in model development and deployment. The highest number in each row is bolded. Cells are shaded if they provide less than half of the total items.

© 2022 Lu JH et al. *JAMA Network Open*.

eTable 3. Requested Metrics

Item Description	# Model Reporting Guidelines requesting	Task	Stage	Reporting Rate
AUROC (c- index)	11	Metrics: Discrimination	Model Development	100.00%
Prognostic Index Plot for Validation Data Set	1	Metrics: Discrimination	Model Development	0.00%
Any direct examination of model output, for example a Plot to Visualize Discrimination	2	Metrics: Discrimination	Model Development	83.33%
Normalized root-mean squared error	1	Metrics: Goodness-of-Fit	Model Development	16.67%
R^2	1	Metrics: Goodness-of-Fit	Model Development	8.33%
Brier Score	1	Metrics: Goodness-of-Fit	Model Development	0.00%
D-statistic	3	Metrics: Goodness-of-Fit	Model Development	0.00%
For survival curves, the log-rank test	1	Metrics: Goodness-of-Fit	Model Development	N/A
Odds Ratio of two different models for comparison	2	Metrics: Goodness-of-Fit	Model Development	0.00%
Calibration Plot	6	Metrics: Calibration	Model Development	0.00%
Survival Curve/Kaplan-Meier Curve superimposition (for Cox models)	2	Metrics: Calibration	Model Development	N/A
PPV	8	Metrics: Classification	Model Development	66.67%
NPV	6	Metrics: Classification	Model Development	16.67%
Sensitivity, ideally at a predefined probability threshold.	9	Metrics: Classification	Model Development	41.67%
Specificity, ideally at a predefined probability threshold.	8	Metrics: Classification	Model Development	8.33%
Full Contingency Table against Reference (includes True/False Positives/Negatives)	2	Metrics: Classification	Model Development	0.00%
True Positive (TP)	1	Metrics: Classification	Model Development	8.33%
True Negative (TN)	1	Metrics: Classification	Model Development	0.00%

© 2022 Lu JH et al. *JAMA Network Open*.

False Positive / False Positive Rate	2	Metrics: Classification	Model Development	16.67%
False Negative / False Negative Rate	2	Metrics: Classification	Model Development	8.33%
False Discovery Rate	1	Metrics: Classification	Model Development	0.00%
False Omission Rate	1	Metrics: Classification	Model Development	0.00%
F score / Dice Coefficient	1	Metrics: Classification	Model Development	0.00%
NNT	1	Metrics: Utility	Utility Assessment	0.00%
Net Benefit (Decision Curve)	3	Metrics: Utility	Utility Assessment	0.00%
Relative Utility (Decision Curve)	1	Metrics: Utility	Utility Assessment	0.00%
Net Reclassification Improvement	5	Metrics: Compare Two Model Discrimination	Model Development	0.00%
Integrated Discrimination Improvement	2	Metrics: Compare Two Model Discrimination	Model Development	0.00%

All items requested, relating to a model performance metric are listed. Reporting Rate indicates the % of the Model Briefs that provided the information requested in the item; N/A means the item did not apply to any Model Briefs. Task and Stage indicate the items' related task and related stage of clinical predictive model development, respectively [13].

© 2022 Lu JH et al. *JAMA Network Open*.

eTable 4. Uniquely Requested Items

**Item Description**	**Requesting Model Reporting Guideline**	**Task**	**Stage**	**Reporting Rate**
How should the model be cited?	Model Cards	Overview	Other: Logistics	0.00%
Is the model regulated or approved by the FDA?	Trust and Value	Overview	Deployed Model: Monitoring	0.00%
Model Name	Model Facts Labels	Overview	Other: Logistics	100.00%
For clinical trials, names and roles of folks involved in clinical trial protocol.	SPIRIT-AI	Overview: Clinical Trial	Other: Logistics	N/A
For clinical trials, names and roles of individuals/groups who oversee.	SPIRIT-AI	Overview: Clinical Trial	Other: Personnel	N/A
For clinical trials, sponsor contact info	SPIRIT-AI	Overview: Clinical Trial	Other: Personnel	N/A
For clinical trials, plans/status of research ethics review approval.	SPIRIT-AI	Overview: Clinical Trial	Other: Logistics	N/A
For clinical trials, plan to communicate or report results to participants and other stakeholders	SPIRIT-AI	Overview: Clinical Trial	Other: Logistics	N/A
For clinical trials, Authorship eligibility guidelines and any intended use of professional writers.	SPIRIT-AI	Overview: Clinical Trial	Other: Personnel	N/A
For clinical trials, Plans for collection, laboratory evaluation, and storage of biological specimens for genetic or molecular analysis in the current trial and for future use in ancillary studies, if applicable.	SPIRIT-AI	Overview: Clinical Trial	Other: Logistics	N/A
It is clear what each data point (i.e. what does a n=1 mean?) of the data set is.	Guidelines	Data Composition	Model Development	100.00%
Clarify if input data is structured (defined like medications) or unstructured (pixels, natural language, time series)	MI-CLAIM	Data Composition: Input	Model Formulation	100.00%
Has there been a check on input features that correlate with protected user categories, which may lead to uninclusive, privacy-breaching or discriminatory results?	ML Test Score	Data Composition: Factors	Model Development: Fairness	8.33%
Report the distribution of severity/stage of disease in those with the target condition	STARD	Data Composition: Output	Model Development	0.00%

© 2022 Lu JH et al. *JAMA Network Open*.

Report the distribution of alternative diagnoses in those without the target condition.	STARD	Data Composition: Output	Model Development	8.33%
Describe if data annotators were given compensation.	Model Cards	Data Collection & Methods	Other: Personnel	0.00%
If there was a time interval and any interventions that occurred between the diagnostic index test and the reference standard, report it.	STARD	Data Collection & Methods: Outcome	Model Development	57.14%
The time interval between the assessment of the predictors and the outcome is appropriate to allow the correct type and representative number of relevant outcomes to be recorded	PROBAST	Data Collection & Methods: Outcome	Model Development	91.67%
Define any strategies for improving and monitoring adherence to interventions.	SPIRIT-AI	Evaluation-specific Study Details: Methods	Prospective Evaluation	N/A
Clarify any retention and follow-up strategies for patients.	SPIRIT-AI	Evaluation-specific Study Details: Methods	Prospective Evaluation	N/A
Plan to communicate clinical protocol amendments to all relevant stakeholders	SPIRIT-AI	Evaluation-specific Study Details: Methods	Other: Logistics	N/A
Changes to clinical trial methods after start of trial	CONSORT-AI	Evaluation-specific Study Details: Methods	Prospective Evaluation	N/A
Guidelines for when / why to stop clinical trial	CONSORT-AI	Evaluation-specific Study Details: Methods	Prospective Evaluation	N/A
Why trial ended or was stopped	CONSORT-AI	Evaluation-specific Study Details: Methods	Prospective Evaluation	N/A

For clinical trials, structure and role of the clinical trial data monitoring committee	SPIRIT-AI	Evaluation-specific Study Details: Methods	Other: Personnel	N/A
For clinical trials, discuss who audits conduct	SPIRIT-AI	Evaluation-specific Study Details: Methods	Other: Personnel	N/A
Ancillary and post-trial care/compensation for those suffering harm	SPIRIT-AI	Evaluation-specific Study Details: Methods	Other: Logistics	N/A
Details on how similar interventions are and relation to concealment	SPIRIT-AI	Evaluation-specific Study Details: Blinding	Prospective Evaluation	N/A
Emergency unblinding procedures	SPIRIT-AI	Evaluation-specific Study Details: Blinding	Prospective Evaluation	N/A
Clinical Outcomes: any changes to definition	CONSORT-AI	Evaluation-specific Study Details: Outcomes	Prospective Evaluation	N/A
Define the results of the clinical outcomes based on definitions.	CONSORT-AI	Evaluation-specific Study Details: Outcomes	Prospective Evaluation	100.00%
Clinical Outcomes: Binary Outcomes show both absolute and relative effect sizes	CONSORT-AI	Evaluation-specific Study Details: Outcomes	Prospective Evaluation	N/A
Provide a check that model training is reproducible	ML Test Score	Model Building	Model Development	8.33%
Check that training/learning objectives for ML are correlated with desired clinical impact metrics	ML Test Score	Model Building	Model Development	0.00%

© 2022 Lu JH et al. *JAMA Network Open*.

How indeterminate model outputs were handled	STARD	Model Summary	Model Formulation	0.00%
Report most predictive features of model	Guidelines	Model Examination	Model Development	8.33%
A check to see if each feature is helping predictive power	ML Test Score	Model Examination	Model Development	58.33%
Disaggregate performance by intersection of subgroups	Model Cards	Model Examination	Model Development: Fairness	0.00%
Report at least 2 distinct model examinations.	MI-CLAIM	Model Examination	Model Development	100.00%
Perform a sensitivity analysis of the model	MI-CLAIM	Model Examination	Model Development	8.33%
Discuss the model examination and performance tradeoffs	MI-CLAIM	Model Examination	Model Development	25.00%
Discuss model reliability under distribution shifts.	MI-CLAIM	Model Examination	Model Development	8.33%
Describe how predictions were calculated in an external validation	TRIPOD	Validation	Model Development	9.09%
Prognostic Index Plot for Validation Data Set	TRIPOD	Metrics: Discrimination	Model Development	0.00%
Normalized root-mean squared error	Guidelines	Metrics: Goodness-of-Fit	Model Development	16.67%
R^2	TRIPOD	Metrics: Goodness-of-Fit	Model Development	8.33%
Brier Score	TRIPOD	Metrics: Goodness-of-Fit	Model Development	0.00%
For survival curves, the log-rank test	CHARMS	Metrics: Goodness-of-Fit	Model Development	N/A
True Positive (TP)	STARD	Metrics: Classification	Model Development	8.33%
True Negative (TN)	STARD	Metrics: Classifica	Developme	0.00%

© 2022 Lu JH et al. *JAMA Network Open*.

		tion	nt	
False Discovery Rate	Model Cards	Metrics: Classification	Model Development	0.00%
False Omission Rate	Model Cards	Metrics: Classification	Model Development	0.00%
F score / Dice Coefficient	MI-CLAIM	Metrics: Classification	Model Development	0.00%
NNT	MI-CLAIM	Metrics: Utility	Utility Assessment	0.00%
Relative Utility (Decision Curve)	TRIPOD	Metrics: Utility	Utility Assessment	0.00%
Description of the clinical activity or annoyance that may be required to make the model, e.g. manually enter info, move to another screen, or otherwise make additional "clicks"?	Trust and Value	Intended Use: Warnings	Use Case	8.33%
A warning on when to stop use of model	Model Facts Labels	Intended Use: Warnings	Use Case	8.33%
Check that no feature costs too much to have (e.g. dependencies, latency, instability, maintenance costs) compared with its added predictive value	ML Test Score	Deployment: Tests: Data	Practical Feasibility	0.00%
Programmatically enforce that input features adhere to meta-level requirements (e.g. deprecated features, protected features).	ML Test Score	Deployment: Tests: Data	Deployed Model: Execution	0.00%
In deployment, a new feature can be added quickly (e.g. within 1-2 months) to the model from ideation.	ML Test Score	Deployment: Tests: Data	Deployed Model: Execution	0.00%
Develop unit tests for input features.	ML Test Score	Deployment: Tests: Data	Deployed Model: Monitoring	0.00%
Monitor input data to ensure that it falls within correct ranges and invariances.	ML Test Score	Deployment: Tests: Data	Deployed Model: Monitoring	0.00%
Perform unit tests of model specification: API usage (e.g. check API calls on a random input) and algorithmic correctness (is it producing the predictions for the correct reasons)?	ML Test Score	Deployment: Tests: Infrastructure	Deployed Model: Monitoring	0.00%

Continuously perform an integration test: a fully automated test that runs regularly and uses the entire pipeline, validating that data and code can successfully move through each stage and that the resulting model performs well.	ML Test Score	Deployment: Tests: Infrastructure	Deployed Model: Monitoring	0.00%
Model allows debugging by step-by-step computation of training/inference on single example	ML Test Score	Deployment: Tests: Infrastructure	Deployed Model: Monitoring	0.00%
Models are tested via a canary process before they enter production serving environments:	ML Test Score	Deployment: Tests: Infrastructure	Deployed Model: Monitoring	0.00%
Report the parts of the models that have been updated and the performance of the updated model	TRIPOD	Deployment: Updating	Deployed Model: Monitoring	16.67%
Every model specification undergoes a code review and is checked in to a repository:	ML Test Score	Deployment: Updating	Deployed Model: Monitoring	0.00%
Check that models can be quickly, easily rolled back in case of emergency.	ML Test Score	Deployment: Updating	Deployed Model: Monitoring	0.00%
Monitor the age of the model and determine how old will affect the staleness of the model.	ML Test Score	Deployment: Monitoring	Deployed Model: Monitoring	0.00%
The deployment team has a line of communication with upstream, dependent data sources and is familiar with new data source changes.	ML Test Score	Deployment: Monitoring	Deployed Model: Monitoring	0.00%
Programmatically check whether data matches invariants in schema and alert when they diverge significantly, tuning a reasonable false positive/false negative point.	ML Test Score	Deployment: Monitoring	Deployed Model: Monitoring	0.00%
Check that training and serving features compute the same values, either by direct comparison of features computed in both systems, or by comparing distributions.	ML Test Score	Deployment: Monitoring	Deployed Model: Monitoring	0.00%
Check degradations in the model computational performance.	ML Test Score	Deployment: Monitoring	Deployed Model: Monitoring	0.00%
Discuss any risk mitigation strategies used during model development.	Model Cards	Ethics	Model Development: Fairness	25.00%
Acknowledge if the model is intended to inform decisions about	Model Cards	Ethics	Use Case	91.67%

© 2022 Lu JH et al. *JAMA Network Open*.

human life or safety.				
Describe any pitfalls in interpreting the model.	Guidelines	Limitations	Model Development	33.33%

All items requested by exactly 1 model reporting guideline are listed. Reporting Rate indicates the % of the Model Briefs that provided the information requested in the item. Task and Stage indicate the items' related task and related stage of clinical predictive model development, respectively [13].

© 2022 Lu JH et al. *JAMA Network Open*.

eTable 5. Epic Model Brief Completion Rates

	EPIC MODEL BRIEFS											
	Deterioration Index	Early Detection of Sepsis	Risk of Unplanned Readmission	Risk of Patient No-Show	Pediatric Risk of Hospital Admission or ED Visit	Risk of Hospital Admission or ED Visit	Inpatient Risk of Falls	Projected Block Utilization	Remaining Length of Stay	Risk of Admission of Heart Failure	Risk of Hospital Admission or ED Visit for Asthma	Risk of Hypertension
**# Reported**	77	68	76	73	53	81	66	55	68	64	62	66
**# Applicable**	166	169	169	170	171	173	171	171	173	172	173	173
**Completion Rate**	46%	40%	45%	43%	31%	47%	39%	32%	39%	37%	36%	38%
**# Reported, excluding performance metrics**	72	61	71	69	48	76	64	49	63	60	58	62
**# Applicable, excluding performance metrics**	140	144	143	144	145	147	145	145	147	146	147	147
**Completion Rate, excluding performance metrics**	51%	42%	50%	48%	33%	52%	44%	34%	43%	41%	39%	42%

A Model Brief's "completion rate" of a given group of items is the number of items reported by the Model Brief divided by the number of items that were applicable to that Model Brief. Cells are colored green if above 50% and yellow if between 25% and 50%.

© 2022 Lu JH et al. *JAMA Network Open*.

eTable 6. Commonly Reported Items

Item Description	Reporting Rate	# Applicable	# Filled	# Model Reporting Guidelines requesting	Task	Stage
Who and how to contact with questions about the model	100.00%	12	12	2	Overview	Other: Personnel
Model Name	100.00%	12	12	1	Overview	Other: Logistics
Date of model development and/or last update	100.00%	12	12	2	Overview	Model Formulation
Model one-line summary	100.00%	12	12	2	Overview	Model Formulation
Scientific / clinical background and rationale for model use (e.g. previous work, clinical role)	100.00%	12	12	6	Overview	Use Case
Specify the type of prediction problem: classification, regression, survival prediction	91.67%	12	11	2	Overview	Model Formulation
Specify whether the data/study was retrospective or prospective.	100.00%	12	12	3	Overview	Model Development
Specify whether the data/study was prognostic or diagnostic?	100.00%	12	12	4	Overview	Model Formulation
Summarize, discuss and interpret results	91.67%	12	11	2	Overview	Other
Specify who (person/organization) built the model	100.00%	12	12	2	Overview	Other: Personnel
Provide any description of the data set (training / study) in question	100.00%	12	12	12	Data Composition	Model Development
For the data set in question, what the sample size is and how it was arrived at, if pre-specified (e.g. Events Per Variable minima)	91.67%	12	11	9	Data Composition	Model Development
It is clear what each data point (i.e. what does a n=1 mean?) of the data set is.	100.00%	12	12	1	Data Composition	Model Development
Describe, list and/or define all input features	100.00%	12	12	7	Data Composition: Input	Model Formulation
Clarify if input data is structured (defined like medications) or unstructured (pixels, natural language, time series)	100.00%	12	12	1	Data Composition: Input	Model Formulation
It is clear if there is a reasonable number of Events per predictor (typically >= 10 or 20)?	91.67%	12	11	4	Data Composition: Input	Model Development

© 2022 Lu JH et al. *JAMA Network Open*.

It is clear if candidate predictors are available at time of intended use of model?	91.67%	12	11	2	Data Composition: Input	Practical Feasibility
Define the output/outcome produced by the model	100.00%	12	12	10	Data Composition: Output	Model Formulation
It is clear whether the outcome is a single or combined endpoint (e.g. cardiovascular disease including heart disease and stroke)	100.00%	12	12	2	Data Composition: Output	Model Development
Define the target population of the data in question (who the model should generalize / apply to?)	100.00%	12	12	8	Study Design/Population	Use Case
Define the specific inclusion/exclusion criteria for participants in data (especially in clinical trials)	100.00%	12	12	9	Study Design/Population	Model Development
Define the specific local area/environment/setting of training data / model deployment.	100.00%	12	12	10	Study Design/Population	Use Case
Define the timeline of data collection. This could, for example, include participant recruitment time, time of predictor measurement, and outcome measurement/followup time.	100.00%	12	12	9	Data Collection & Methods	Model Development
Details of treatments received by participants, if relevant. (NOT studying specific interventions for patients, just what treatments they may be receiving already)	90.91%	11	10	2	Data Collection & Methods	Model Development
Consistent Outcome Definition and Measurement for all patients	100.00%	12	12	3	Data Collection & Methods: Outcome	Model Development
Predictors Not Part of Outcome (e.g. in panel or consensus diagnosis)	100.00%	12	12	2	Data Collection & Methods: Outcome	Model Development
The time interval between the assessment of the predictors and the outcome is appropriate to allow the correct type and representative number of relevant outcomes to be recorded	91.67%	12	11	1	Data Collection & Methods: Outcome	Model Development
Define evaluation outcomes for intervention assessment.	100.00%	1	1	3	Evaluation-specific Study Details: Outcome	Prospective Evaluation

© 2022 Lu JH et al. *JAMA Network Open*.

					s	
Define the results of the clinical outcomes based on definitions.	100.00%	1	1	1	Evaluation-specific Study Details: Outcomes	Prospective Evaluation
How data was preprocessed (data cleaning, predictor transformation, outlier removal, predictor coding)	100.00%	12	12	10	Preprocessing and Data Cleaning	Model Development
Clarify the type of final model to be used	91.67%	12	11	9	Model Summary	Model Formulation
Report at least 2 distinct model examinations.	100.00%	12	12	1	Model Examination	Model Development
Clarify what type of validation is done, whether internal or external	100.00%	12	12	11	Validation	Model Development
Describe internal validation strategy to account for model optimism (e.g. cross-validation, bootstrapping, data splitting))	100.00%	11	11	11	Validation	Model Development
Mention what performance measures are used	100.00%	12	12	13	Metrics	Model Development
AUROC (c- index)	100.00%	11	11	11	Metrics: Discrimination	Model Development
Describe how the ML model is supposed to be used in clinical context	100.00%	12	12	11	Intended Use	Use Case
Suggest ways the ML model could impact clinical care (no study needed, it's okay if this is speculative)	100.00%	12	12	6	Intended Use	Utility Assessment
Specify who will use the ML model.	100.00%	12	12	5	Intended Use: User	Use Case
Acknowledge if the model is intended to inform decisions about human life or safety.	91.67%	12	11	1	Ethics	Use Case

All items reported by 90% or more of applicable Model Briefs are listed. Reporting Rate indicates the % of the Model Briefs that provided the information requested in the item. Task and Stage indicate the items' related task and related stage of clinical predictive model development, respectively [13].

© 2022 Lu JH et al. *JAMA Network Open*.

eTable 7. Rarely Reported Items

Item Description	Reporting Rate	# Applicable	# Filled	# Model Reporting Guidelines requesting	Task	Stage
How should the model be cited?	0.00%	11	0	1	Overview	Other: Logistics
Is the model regulated or approved by the FDA?	0.00%	12	0	1	Overview	Deployed Model: Monitoring
Specify who funded / supported the study and clarify any conflicts of interest	0.00%	10	0	4	Overview	Other: Personnel
Information on how to access the data used	0.00%	12	0	4	Data Composition	Other: Logistics
Provide statistics on the amount of missing data there is.	8.33%	12	1	5	Data Composition	Model Development
Has there been a check on input features that correlate with protected user categories, which may lead to uninclusive, privacy-breaching or discriminatory results?	8.33%	12	1	1	Data Composition: Factors	Model Development: Fairness
Report the distribution of severity/stage of disease in those with the target condition	0.00%	11	0	1	Data Composition: Output	Model Development
Report the distribution of alternative diagnoses in those without the target condition.	8.33%	12	1	1	Data Composition: Output	Model Development
Flow chart of how participants were interacted/assigned/followed up with in the study (especially in clinical trials)	0.00%	12	0	5	Data Collection & Methods	Model Development
Describe if data annotators were given compensation.	0.00%	12	0	1	Data Collection & Methods	Other: Personnel
Describe the interobserver/inter-study agreement on data coding, and if there was any standardization effort.	0.00%	12	0	3	Data Collection & Methods	Model Development
Blinding of Data Collectors/Predictor Assessors to outcomes, if done	0.00%	9	0	4	Data Collection & Methods: Input	Model Development
Blinding of Outcome Assessors to predictors of the model, if done	0.00%	9	0	7	Data Collection & Methods: Outcome	Model Development

© 2022 Lu JH et al. *JAMA Network Open*.

Define who obtains consent to data collection	0.00%	12	0	2	Data Collection & Methods: Consent/Privacy for Data	Other: Personnel
Define what form or measures are taken to ensure informed consent for patients.	0.00%	12	0	2	Data Collection & Methods: Consent/Privacy for Data	Other: Logistics
Define what provisions are taken for participant data use in followup studies.	0.00%	12	0	2	Data Collection & Methods: Consent/Privacy for Data	Other: Logistics
Define how confidentiality and privacy will be ensured for participants' data.	0.00%	12	0	3	Data Collection & Methods: Consent/Privacy for Data	Other: Logistics
Provide a check that model training is reproducible	8.33%	12	1	1	Model Development	Model Development
Check that training/learning objectives for ML are correlated with desired clinical impact metrics	0.00%	12	0	1	Model Development	Model Development
Describe which features were allowed interactions.	0.00%	12	0	2	Model Development	Model Development
Provide confidence intervals, statistical significance, or some other handling of uncertainty and variability in model performance metrics	0.00%	12	0	10	Model Performance and Comparison	Model Development
Provide sufficient information to enable reproducibility/replication	0.00%	12	0	7	Model Development	Other: Logistics
How indeterminate model outputs were handled	0.00%	12	0	1	Model Summary	Model Formulation
Report model coefficients (regression) or saliency map	8.33%	12	1	7	Model Examination	Model Development
Report most predictive features of model	8.33%	12	1	1	Model Examination	Model Development

© 2022 Lu JH et al. *JAMA Network Open*.

Describe cases where the model had high or low performance error	8.33%	12	1	2	Model Examination	Model Development
Disaggregate performance by intersection of subgroups	0.00%	12	0	1	Model Examination	Model Development: Fairness
Perform a sensitivity analysis of the model	8.33%	12	1	1	Model Examination	Model Development
Analyze the model's performance errors	0.00%	12	0	2	Model Examination	Model Development
Discuss model reliability under distribution shifts.	8.33%	12	1	1	Model Examination	Model Development
Describe how predictions were calculated in an external validation	9.09%	11	1	1	Validation	Model Development
Prognostic Index Plot for Validation Data Set	0.00%	10	0	1	Metrics: Discrimination	Model Development
R^2	8.33%	12	1	1	Metrics: Goodness-of-Fit	Model Development
Brier Score	0.00%	12	0	1	Metrics: Goodness-of-Fit	Model Development
D-statistic	0.00%	1	0	3	Metrics: Goodness-of-Fit	Model Development
Odds Ratio of two different models for comparison	0.00%	12	0	2	Metrics: Goodness-of-Fit	Model Development
Calibration Plot	0.00%	12	0	6	Metrics: Calibration	Model Development
Specificity, ideally at a predefined probability threshold.	8.33%	12	1	8	Metrics: Classification	Model Development
Full Contingency Table against Reference (includes True/False Positives/Negatives)	0.00%	12	0	2	Metrics: Classification	Model Development
True Positive (TP)	8.33%	12	1	1	Metrics: Classification	Model Development
True Negative (TN)	0.00%	12	0	1	Metrics: Classification	Model Development

© 2022 Lu JH et al. *JAMA Network Open*.

False Negative / False Negative Rate	8.33%	12	1	2	Metrics: Classification	Model Development
False Discovery Rate	0.00%	12	0	1	Metrics: Classification	Model Development
False Omission Rate	0.00%	12	0	1	Metrics: Classification	Model Development
F score / Dice Coefficient	0.00%	12	0	1	Metrics: Classification	Model Development
NNT	0.00%	12	0	1	Metrics: Utility	Utility Assessment
Net Benefit (Decision Curve)	0.00%	12	0	3	Metrics: Utility	Utility Assessment
Relative Utility (Decision Curve)	0.00%	12	0	1	Metrics: Utility	Utility Assessment
Net Reclassification Improvement	0.00%	12	0	5	Metrics: Compare Two Model Discrimination	Model Development
Integrated Discrimination Improvement	0.00%	12	0	2	Metrics: Compare Two Model Discrimination	Model Development
Compare model's performance to that of a baseline model, with statistical significance.	0.00%	12	0	2	Comparison Against Baseline Model	Model Development
Specify what directions, explanations and other user-facing materials there will be with the model.	0.00%	12	0	9	Intended Use: User	Use Case
Description of the clinical activity or annoyance that may be required to make the model, e.g. manually enter info, move to another screen, or otherwise make additional "clicks"?	8.33%	12	1	1	Intended Use: Warnings	Use Case
A warning on when to stop use of model	8.33%	12	1	1	Intended Use: Warnings	Use Case
Guidance on specific technical issues to address for integration of the model into your care setting, e.g. hardware, cloud, software or computing environment needs.	8.33%	12	1	2	Deployment	Practical Feasibility

© 2022 Lu JH et al. *JAMA Network Open*.

How private data from participants on which the model is deployed is protected. (this is deployment data, not training data)	0.00%	12	0	3	Deployment: Tests: Data	Practical Feasibility
Check that no feature costs too much to have (e.g. dependencies, latency, instability, maintenance costs) compared with its added predictive value	0.00%	12	0	1	Deployment: Tests: Data	Practical Feasibility
Programmatically enforce that input features adhere to meta-level requirements (e.g. deprecated features, protected features).	0.00%	12	0	1	Deployment: Tests: Data	Deployed Model: Execution
In deployment, a new feature can be added quickly (e.g. within 1-2 months) to the model from ideation.	0.00%	12	0	1	Deployment: Tests: Data	Deployed Model: Execution
Develop unit tests for input features.	0.00%	12	0	1	Deployment: Tests: Data	Deployed Model: Monitoring
Monitor input data to ensure that it falls within correct ranges and invariances.	0.00%	12	0	1	Deployment: Tests: Data	Deployed Model: Monitoring
Perform unit tests of model specification: API usage (e.g. check API calls on a random input) and algorithmic correctness (is it producing the predictions for the correct reasons)?	0.00%	12	0	1	Deployment: Tests: Infrastructure	Deployed Model: Monitoring
Continuously perform an integration test: a fully automated test that runs regularly and uses the entire pipeline, validating that data and code can successfully move through each stage and that the resulting model performs well.	0.00%	12	0	1	Deployment: Tests: Infrastructure	Deployed Model: Monitoring
Model allows debugging by step-by-step computation of training/inference on single example	0.00%	12	0	1	Deployment: Tests: Infrastructure	Deployed Model: Monitoring
Models are tested via a canary process before they enter production serving environments:	0.00%	12	0	1	Deployment: Tests: Infrastructure	Deployed Model: Monitoring
Every model specification undergoes a code review and is checked in to a repository:	0.00%	12	0	1	Deployment: Updating	Deployed Model: Monitoring
Check that models can be quickly, easily rolled back in case of emergency.	0.00%	12	0	1	Deployment: Updating	Deployed Model: Monitoring
Monitor regressions in prediction quality in newer data.	8.33%	12	1	3	Deployment: Monitoring	Deployed Model: Monitoring

© 2022 Lu JH et al. *JAMA Network Open*.

Monitor the age of the model and determine how old will affect the staleness of the model.	0.00%	12	0	1	Deployment: Monitoring	Deployed Model: Monitoring
The deployment team has a line of communication with upstream, dependent data sources and is familiar with new data source changes.	0.00%	12	0	1	Deployment: Monitoring	Deployed Model: Monitoring
Programmatically check whether data matches invariants in schema and alert when they diverge significantly, tuning a reasonable false positive/false negative point.	0.00%	12	0	1	Deployment: Monitoring	Deployed Model: Monitoring
Monitor numerical stability of model, including NaNs and infinities in model components/weights or predictions.	0.00%	12	0	2	Deployment: Monitoring	Deployed Model: Monitoring
Check that training and serving features compute the same values, either by direct comparison of features computed in both systems, or by comparing distributions.	0.00%	12	0	1	Deployment: Monitoring	Deployed Model: Monitoring
Check degradations in the model computational performance.	0.00%	12	0	1	Deployment: Monitoring	Deployed Model: Monitoring
Acknowledge any multiplicity of analyses or comparisons which may cause spurious signals.	0.00%	12	0	2	Limitations	Model Development

All items reported by 10% or less of applicable Model Briefs are listed. Reporting Rate indicates the % of the Model Briefs that provided the information requested in the item. Task and Stage indicate the items' related task and related stage of clinical predictive model development, respectively [13].

© 2022 Lu JH et al. *JAMA Network Open*.

eTable 8. Low Consensus Items

Item Description	# Consensus	Task	Stage	Reporting Rate	# Model Reporting Guidelines requesting
Who and how to contact with questions about the model	0	Overview	Other: Personnel	100.0%	2
Summarize, discuss and interpret results	0	Overview	Other	91.7%	2
Specify who funded / supported the study and clarify any conflicts of interest	0	Overview	Other: Personnel	0.0%	4
It is clear if candidate predictors are available at time of intended use of model?	0	Data Composition: Input	Practical Feasibility	91.7%	2
Report the distribution of severity/stage of disease in those with the target condition	0	Data Composition: Output	Model Development	0.0%	1
Report the distribution of alternative diagnoses in those without the target condition.	0	Data Composition: Output	Model Development	8.3%	1
Describe the design of the study that was used to collect the data.	0	Study Design/Population	Model Development	83.3%	5
Describe how participants were enrolled or recruited into the data.	0	Data Collection & Methods	Model Development	58.3%	3
Define the timeline of data collection. This could, for example, include participant recruitment time, time of predictor measurement, and outcome measurement/followup time.	0	Data Collection & Methods	Model Development	100.0%	9
Details of treatments received by participants, if relevant. (NOT studying specific interventions for patients, just what treatments they may be receiving already)	0	Data Collection & Methods	Model Development	90.9%	2
Overview of data collection, annotation, and quality process	0	Data Collection & Methods	Model Development	66.7%	8
Describe the annotation process of the input data, including who annotated the input data, what instructions they were given, and what expertise was needed.	0	Data Collection & Methods: Input	Model Development	18.2%	4

© 2022 Lu JH et al. *JAMA Network Open*.

Blinding of Data Collectors/Predictor Assessors to outcomes, if done	0	Data Collection & Methods: Input	Model Development	0.0%	4
Describe the annotation process of the output data, including who annotated the output data, what instructions they were given, and what expertise was needed.	0	Data Collection & Methods: Outcome	Model Development	27.3%	7
Blinding of Outcome Assessors to predictors of the model, if done	0	Data Collection & Methods: Outcome	Model Development	0.0%	7
Consistent Outcome Definition and Measurement for all patients	0	Data Collection & Methods: Outcome	Model Development	100.0%	3
Reference standard for determining the outcome, if used	0	Data Collection & Methods: Outcome	Model Development	44.4%	3
If there was a time interval and any interventions that occurred between the diagnostic index test and the reference standard, report it.	0	Data Collection & Methods: Outcome	Model Development	57.1%	1
Predictors Not Part of Outcome (e.g. in panel or consensus diagnosis)	0	Data Collection & Methods: Outcome	Model Development	100.0%	2
The time interval between the assessment of the predictors and the outcome is appropriate to allow the correct type and representative number of relevant outcomes to be recorded	0	Data Collection & Methods: Outcome	Model Development	91.7%	1
How data was preprocessed (data cleaning, predictor transformation, outlier removal, predictor coding)	0	Preprocessin g and Data Cleaning	Model Development	100.0%	10
It is clear if categorical predictors have been dichotomized or categorized prior to model development.	0	Preprocessin g and Data Cleaning	Model Development	83.3%	2
Were all enrolled participants included in the analysis? (Not doing so leads to risk of bias. Number of participants included in each analysis and whether	0	Preprocessin g and Data Cleaning	Model Development	58.3%	3

© 2022 Lu JH et al. *JAMA Network Open*.

the analysis was by original assigned groups)					
If feature selection involved computing univariate associations between input features and outcomes (not recommended), document this.	0	Preprocessing and Data Cleaning	Model Development	18.2%	4
Describe which features were allowed interactions.	0	Model Building	Model Development	0.0%	2
If a survival function is used, provide the baseline survival function.	0	Model Summary	Model Formulation	N/A	2
Report some examination of what the model is doing beyond the primary performance measure.	0	Model Examination	Model Development	75.0%	7
Prognostic Index Plot for Validation Data Set	0	Metrics: Discrimination	Model Development	0.0%	1
D-statistic	0	Metrics: Goodness-of-Fit	Model Development	0.0%	3
For survival curves, the log-rank test	0	Metrics: Goodness-of-Fit	Model Development	N/A	1
Survival Curve/Kaplan-Meier Curve superimposition (for Cox models)	0	Metrics: Calibration	Model Development	N/A	2
Acknowledge if the model is intended to inform decisions about human life or safety.	0	Ethics	Use Case	91.7%	1
Discuss any limitations and caveats of the study.	0	Limitations	Use Case	83.3%	6
Discuss if or why well-known predictors were omitted from the model.	0	Limitations	Model Development	25.0%	2

All items with no consensus among the reviewers are listed. Reporting Rate indicates the % of the Model Briefs that provided the information requested in the item; N/A means the item did not apply to any Model Briefs. Task and Stage indicate the items' related task and related stage of clinical predictive model development, respectively [13].

© 2022 Lu JH et al. *JAMA Network Open*.

eAppendix. Code for Methods, Grading of Model Briefs, Adjudication, and Analysis

Code for methods, including merging of guidelines, deduplication of items, mapping of items onto stages of model development and tasks, grading of Model Briefs, adjudication, and analysis. The reporting rate of every Item can be found in the "Item Summary" sheet. This can be accessed through either of these links: https://tinyurl.com/modelreportsheet or https://docs.google.com/spreadsheets/d/1o26raifyZ_3FzvUOJYu39xoPcsezSjWTdDBm32IVS-8/edit?usp=sharing

© 2022 Lu JH et al. *JAMA Network Open*.

eReferences

1. *Cognitive Computing Model Brief: Deterioration Index*. Epic; 2021. https://galaxy.epic.com/?#Browse/page=1!68!50!3883949

2. *Cognitive Computing Model Brief: Early Detection of Sepsis*. Epic; 2016. https://galaxy.epic.com/?#Browse/page=1!68!50!3289911

3. *Cognitive Computing Model Brief: Risk of Unplanned Readmission (Version 2)*. Epic; 2020. https://galaxy.epic.com/Redirect.aspx?DocumentID=100051822

4. *Cognitive Computing Model Brief: Risk of Patient No-Show (Version 2)*. Epic; 2021. https://galaxy.epic.com/Redirect.aspx?DocumentID=100020266

5. *Cognitive Computing Model Brief: Pediatric Hospital Admissions and ED Visits*. Epic; 2018. https://galaxy.epic.com/Redirect.aspx?DocumentID=3763630&Version=Epic%202018

6. *Cognitive Computing Model Brief: Risk of Hospital Admission or ED Visit (Version 2)*. Epic; 2020. https://galaxy.epic.com/Redirect.aspx?DocumentID=100045918

7. *Cognitive Computing Model Brief: Inpatient Risk of Falls*. Epic; 2020. https://galaxy.epic.com/?#Browse/page=1!68!50!100014430

8. *Cognitive Computing Model Brief: Projected Block Utilization*. Epic; 2018. https://galaxy.epic.com/Redirect.aspx?DocumentID=100014389

9. *Cognitive Computing Model Brief: Remaining Length of Stay*. Epic; 2017. https://galaxy.epic.com/Redirect.aspx?DocumentID=3364171&Version=Epic%202018

10. *Cognitive Computing Model Brief: Hospital Admissions for Heart Failure*. Epic; 2017. https://galaxy.epic.com/Redirect.aspx?DocumentID=3706332&Version=Epic%202018

11. *Cognitive Computing Model Brief: Hospital Admissions and ED Visits for Asthma*. Epic; 2017. https://galaxy.epic.com/Redirect.aspx?DocumentID=3587370

12. *Cognitive Computing Model Brief: Hypertension*. Epic; 2016. https://galaxy.epic.com/?#Browse/page=1!68!50!3479172

13. Jung K, Kashyap S, Avati A, et al. A framework for making predictive models useful in practice. *J Am Med Inform Assoc*. 2021;28(6):1149-1158.

© 2022 Lu JH et al. *JAMA Network Open*.
